# Preclinical safety and burn wound healing activity of “Novostron”, a novel topical iodine-based therapeutic

**DOI:** 10.1371/journal.pone.0338837

**Published:** 2025-12-12

**Authors:** Nailya Ibragimova, Arailym Aitynova, Seitzhan Turganbay, Marina Lyu, Alexandr Ilin, Tamari Gapurkhaeva, Galina Ponomareva, Karina Vassilyeva, Diana Issayeva, Amirkan Azembayev, Serzhan Mombekov, Aralbek Rsaliyev, Nurgul Sikhayeva, Yergali Abduraimov, Saki Raheem

**Affiliations:** 1 JSC Scientific Center for Anti-Infectious Drugs, Almaty, Kazakhstan; 2 School of Pharmacy, S.D. Asfendiyarov Kazakh National Medical University, Almaty, Kazakhstan; 3 Research Institute for Biological Safety Problems, Gvardeisky, Kazakhstan; 4 JSC National Holding “Qazbiopharm”, Astana, Kazakhstan; 5 School of Life Sciences, University of Westminster, London, United Kingdom; Al-Azhar University Faculty of Science for Boys in Cairo, EGYPT

## Abstract

Iodine-based antiseptics are essential in wound care but are often limited by cytotoxicity, instability, and rapid iodine release. Novostron is a novel polymer–iodine complex incorporating dextrin, polyvinyl alcohol, and metal ions, designed to enable controlled iodine release. Structural integrity and composition were confirmed by ¹H and ^13^C NMR spectroscopy and physicochemical analysis, indicating a molecular weight of ~9500 g/mol, a pH of 4.23, and an iodine content of 8.13%. Pharmacokinetic analysis in rabbits demonstrated that following a single dermal application, systemic iodine absorption was minimal, with peak blood iodine concentrations remaining within physiological limits and rapid elimination within 24 hours. Evaluation of thyroid function revealed no significant changes in serum T₃, T₄, or TSH levels compared with those of the controls, confirming that topical application of Novostron does not disrupt thyroid homeostasis. In compliance with OECD guidelines in rabbits, guinea pigs, and rats, Novostron showed no signs of dermal irritation, skin sensitization, or systemic toxicity (LD₅₀ > 2000 mg/kg). In a rat cotton pellet granuloma model, Novostron significantly reduced the inflammatory mass (23.65% inhibition), supporting its anti-inflammatory potential. In a murine burn model, Novostron accelerated wound contraction (25.95% at day 10), increased epidermal thickness, and enhanced collagen deposition (~44%), outperforming controls and matching or exceeding betadine. These findings suggest that Novostron promotes tissue repair by modulating inflammation. Overall, Novostron demonstrated a favourable preclinical safety and efficacy profile, and its polymer–iodine composition, which enables controlled release and localized activity highlights its potential as a promising topical therapeutic. However, the study was limited to animal models and short-term observation; further long-term and clinical investigations are needed to confirm its translational potential in human wound healing.

## 1 Introduction

Infectious diseases remain a major global health challenge, further compounded by the rise of antimicrobial resistance. This has intensified efforts to develop alternative therapeutic approaches that are both effective and less likely to drive resistance. Topical antimicrobial agents have regained interest due to their localized action, reduced systemic exposure, and lower potential for promoting resistance. Among these, iodine-based compounds stand out for their long-established, broad-spectrum antimicrobial efficacy and sustained activity against various pathogens [1,2].

Iodine exhibits potent antimicrobial activity through multiple mechanisms, including the disruption of electron transport, inhibition of protein synthesis, and denaturation of nucleic acids, ultimately leading to the death of microbial cells [3]. Although iodine-based formulations such as povidone–iodine (PVP-I) are recognized for their broad antimicrobial activity and the absence of microbial resistance [4], they still present certain drawbacks, including rapid iodine volatilization, instability in aqueous media, and occasional local irritation. Therefore, the development of new iodine–polymer complexes such as Novostron aims to improve the stability, sustained release, and safety profile of iodine without compromising its antimicrobial potency.

In pharmaceutical formulations, iodine exists as molecular iodine (I₂), iodide (I⁻), and polyiodide complexes (e.g., I₃ ⁻ , I₅⁻), which can be stabilized within polymeric carriers, such as polyvinylpyrrolidone (PVP), forming iodophors like PVP-I₂ that allow for controlled release [5,6].

Given the need for advanced iodine delivery platforms to overcome microbial adaptation, interest has grown in next-generation iodine complexes that ensure both efficacy and stability. A formulation incorporating dextrin, PVA, and multivalent metal salts promotes stable polyiodide complexation and sustained iodine release [7,8], potentially limiting adaptive microbial responses and enhancing local bioavailability compared to conventional iodophors.

Building on this foundation, a novel iodine-based drug, “Novostron”, was developed for external application. Given its intended dermal application, it is essential to evaluate the safety of Novostron, particularly regarding potential skin irritation, skin sensitization and acute dermal toxicity. These preclinical assessments are crucial for ensuring the safe therapeutic use and identifying any adverse reactions that may occur during topical administration [9]. As the body’s largest and most exposed organ, the skin is a protective barrier but remains vulnerable to chemical agents [10]. Therefore, topical formulations must be rigorously evaluated to prevent irritation or allergic responses [11].

Skin sensitization is an immune-mediated reaction that begins with the chemical modification of skin proteins, followed by antigen presentation by Langerhans cells to naïve T-cells in the lymph nodes [12]. Upon re-exposure, this sensitization cascade can trigger inflammation and tissue damage [12]. Hence, comprehensive testing for irritation and sensitization is fundamental in the development of new dermal therapeutics. Animal models, such as guinea pigs and rabbits, are widely used in dermatotoxicology to assess sensitization’s induction and elicitation phases and evaluate the systemic effects of single-dose dermal exposure [13].

This study aimed to characterize the safety and therapeutic potential of the novel topical formulation, Novostron. We conducted preclinical evaluations of its acute dermal toxicity, skin irritation, and sensitization potential in vivo. Additionally, we assessed its anti-inflammatory and wound-healing properties to determine its suitability as a safe and effective topical antimicrobial agent.

## 2 Materials and methods

### 2.1 Analytical procedures and physicochemical characterization

#### 2.1.1 Chemicals and reagents.

The following reagents were used for chemical synthesis and studies of physicochemical properties: potato starch 98% (JSC Rogoznitsky Starch Plant, Belarus), polyvinyl alcohol 99.8% (PVA) with molecular mass of 31000 (Sigma Aldrich, Germany), magnesium hexahydrate chloride, sodium chloride, hydrochloric acid, sodium hydroxide (JSC Chemical Reagents, Russia), calcium chloride hexahydrate 99% (Karpov Chemical Plant, Russia), lithium chloride 99% (Sigma-Aldrich, Germany), 10% albumin solution (LLC Biopharma Plasma, Ukraine), crystalline iodine, potassium iodide (G. Amphray Lab., India), purified water from water purification system UltraClear TWF (GmbH, Germany), sodium thiosulfate 0.1 N (Fixanal, Uralcheminvest, Russia), silver nitrate 0.1 N (Fixanal), nitric acid 0.1 N (Fixanal) and iodine 0.1 N (Fixanal), Deuterium oxide (D₂O; Sigma-Aldrich, USA). Deionized water with a resistivity of 18.2MΩ-cm was used throughout the experiments. Standard buffer solutions (pH 4.0, 7.0 and 10.0) were obtained from Reagecon Diagnostics Ltd (Ireland) for pH calibration and measurement.

#### 2.1.2 Synthesis of the formulation.

Novostron, an original pharmaceutical formulation developed by the Laboratory of New Substances and Materials (JSC “Scientific Center for Anti-Infectious Drugs,” Almaty, Kazakhstan), was synthesized through a multi-step process involving starch hydrolysis, neutralization, and complexation.

In a jacketed reactor (R1), 10 L of purified water was heated to 120 °C under pressure (stirred at 70 rpm), and 210 mL of HCl was added at 90 °C. A starch suspension was transferred from Reactor R2, followed by rinsing with purified water. Hydrolysis proceeded for 25 minutes at ≥88 °C, as monitored by the reduction in sugar content. After neutralization with 210 mL NaOH, PVA was added from Reactor R1, and sodium and calcium salts were introduced from Reactor R4. After cooling to 43 °C, lithium and magnesium chlorides were added from Reactor R3. The pH was adjusted to 4.5 with NaOH, and the mixture was cooled to 25 °C before adding potassium triiodide from Reactor R5. The emergence of a stable blue color indicated the successful formation of an iodine–dextrin inclusion complex, where I₃ ⁻ anions are embedded within amylose helices.

The synthesis was performed using defined concentrations of elemental iodine (8.2 mg, 0.032 mmol), potassium iodide (12.1 mg, 0.073 mmol), dextrin (130 mg, 0.806 mmol anhydroglucose units) and PVA (3.00 mg, 9.68 x 10^−5^ mmol), alongside lithium chloride (0.10 mg, 0.0024 mmol), sodium chloride (8.00 mg, 0.137 mmol), calcium chloride (0.50 mg, 0.0045 mmol), and magnesium chloride (0.50 mg, 0.0052 mmol). The reaction was carried out at room temperature for 30 minutes with constant stirring to ensure complete dissolution. The resulting mixture was then filtered to remove any undissolved particulates and stored at room temperature.

The active ingredients of Novostron are iodine (I_2_), 8.2 mg/mL, and potassium iodide (KI), 12.1 mg/mL.


$${\text{I}}_{\text{2}}{+\;\text{KI}=\text{KI}}_{\text{3}}$$ (1)

Given the molar masses of molecular, 253.8g/mol for I_2_ (126.9 × 2) and 166.0 g/mol for KI (39.1 + 126.9), the amount of I_2_ in 8.2 mg (0.0082 g) is:


$$n\;\left(I_{\text{2}}\right)=\frac{\text{0}.\text{0082}}{\text{253}.\text{8}}=\text{3}.\text{2}\times \;{\text{1}\text{0}}^{-\text{5}}$$ (2)

And the amount of KI is 12.1 mg (0.0121 g):


$$n\;(KI)=\frac{\text{0}.\text{0}\text{1}\text{2}\text{1}}{\text{166}.\text{0}}=\text{7}.\text{3}\;\times \;{\text{1}\text{0}}^{-\text{5}}$$ (3)

Since KI is in excess, molecular iodine (I_2_) is the limiting reagent and is fully consumed. The equimolar amount of KI is required to bind 3.23 × 10^−5^ mol of I_2_, which corresponds to:


$$m\;(KI)=\text{3}.\text{23}\times \;{\text{1}\text{0}}^{-\text{5}}\;\times \text{1}\text{6}\text{6}.\text{0}=\text{5}.\text{3}\text{6}$$ (4)

Based on calculated molarities, the I₂:KI:dextrin: PVA ratio was approximately 1:2:24:2, ensuring complete stabilization of iodine as I₃⁻ and formation of stable iodine-polymer inclusion complexes.

To describe the coordination structure of the iodine–dextrin complex in Novostron, we propose an empirical composition of:


$$\;{\left({\text{C}}_{\text{6}}{\text{H}}_{\text{1}\text{0}}{\text{O}}_{\text{5}}\right)}_{\text{4}\;\text{9}}\cdot \text{2Li}{\text{I}}_{\text{3}}\cdot \text{Mg}{\left({\text{I}}_{\text{3}}\right)}_{\text{2}}$$

This reflects a representative supramolecular unit, based on the estimated degree of dextrin polymerisation (~49 glucose residues). It illustrates the encapsulation of polyiodine species (e.g., I₃ ⁻ , I₅⁻) within the V-type helical cavities of dextrin, coordinated by lithium and magnesium ions. This formula does not represent a fixed stoichiometric compound, but rather a model derived from input mass ratios, elemental analysis, and coordination patterns inferred from ¹H and ^13^C NMR spectroscopy.

This model supports the interpretation of Novostron as a polyiodide-dextrin network stabilized by multivalent cations and polymeric ligands.

#### 2.1.3 pH measurement.

The pH of the formulation was determined potentiometrically, following the State Pharmacopeia of the Republic of Kazakhstan [14]. A digital pH meter was calibrated using standard buffer solutions at pH levels of 4.0, 7.0, and 10.0, maintained at a temperature of 20 °C. Each sample was placed in a 30 mL tube, and the electrode was immersed in the solution until the reading stabilized.

#### 2.1.4 Viscosity and density.

Following pharmacopeial procedures, kinematic viscosity was measured using a VPZh-4m Ostwald capillary viscometer under standard conditions [14]. Relative density was determined using a calibrated pycnometer at 20 °C. These parameters were evaluated to characterize the formulation’s rheological properties and to assess their potential impact on drug release kinetics in a liquid dosage form.

#### 2.1.5 Determination of free iodine and potassium iodide content.

The free iodine content was quantified by iodometric titration using 0.1 M sodium thiosulfate with starch as an indicator. A weighed portion (0.1–0.3 g) was dissolved in 20 mL of purified water, and titrated until the brown color disappeared. Starch was then added, and titration continued to the endpoint. According to the pharmacopeia, 1 mL of 0.1 M sodium thiosulfate corresponds to 12.69 mg of iodine.

For potassium iodide determination, 5 mL of 0.1 N nitric acid was added to the post-titration mixture. The solution was then titrated potentiometrically with 0.05 M silver nitrate using a Metrohm ionomer (Switzerland) equipped with a combined platinum electrode and an Ag/AgCl internal reference electrode (Sigma-Aldrich, USA). The equivalence point was determined from the inflection point of the potential vs. volume curve. According to the pharmacopeia, 1 mL of 0.1 M AgNO₃ corresponds to 16.6 mg of KI [14].

#### 2.1.6 ^1^H and ^13^C NMR-spectroscopy.

¹H and ^13^C NMR spectra were recorded on a superconducting Fourier-transform NMR spectrometer (JNM-ECA500, JEOL). Operating frequencies were 500 MHz for ¹H and 125 MHz for ^13^C nuclei. Prior to data acquisition, automatic tuning, shimming, and adjustments of magnetic field homogeneity were performed following standard JEOL protocols. Residual solvent peaks were used for field locking and as internal chemical-shift references.

Samples were dissolved in D₂O for ¹H NMR and in D₂O or DMSO-d₆ for ^13^C NMR, depending on solubility, at concentrations of 20–50 mg/mL. Measurements were conducted at 25 ± 1 °C. Solvent-signal suppression was applied using standard pulse sequences when necessary. Acquisition parameters for ¹H NMR included a spectral width of 10,000 Hz, a data size of 32,768 points, and a minimum of 16 scans. For ^13^C NMR, the spectral width was 30,000 Hz, with 64,000 data points, and 2,000–5,000 scans depending on the signal intensity.

Exponential line broadening (0.3 Hz for ¹H and 1 Hz for ^13^C) was applied prior to Fourier transformation. Manual Phase and baseline corrections were performed using TopSpin software. The sample concentration and pH were adjusted as needed to improve spectral quality.

To minimize the potential redox decomposition of iodine-containing components, spectra were acquired in sealed tubes under an argon atmosphere with minimal exposure to light. Chemical-shift stability was evaluated in D₂O and DMSO-d₆ to assess solvent effects and confirm reproducibility. Spectra were examined for impurities, side peaks, and possible degradation products.

Two-dimensional NMR experiments (COSY, HSQC, and HMBC) were performed to aid in signal assignment and to characterize structural features of the dextin-iodine complex.

### 2.2 *In vivo* studies, randomization and blinding

Animals were assigned to experimental groups using a predefined randomization procedure. Each animal received a unique identification number upon enrollment, after which group allocation was determined using a computer-generated random sequence in Microsoft Excel (using the RAND function).

Allocation concealment and blinding were maintained throughout all experiments. Treatment formulations were prepared and labeled with anonymized study codes by a separate staff member to ensure that investigators responsible for animal handling, wound treatment, and data analysis remained blinded to the group assignment. Outcome assessments, including clinical observation, wound measurement, photographic analysis, microbiology, and histopathology, were performed by investigators blinded to treatment allocation. Histological slides and photographic files were coded and analyzed without knowledge of group identity, and the decoding was performed only after the completion of primary data analysis. No unblinding events occurred during the study.

### 2.3 ARRIVE 2.0 compliance and animal welfare

All animal procedures were conducted in compliance with Directive 2010/63/EU, the Law of the Republic of Kazakhstan “On Responsible Treatment of Animals” (No. 45-r, March 4, 2022), and ARRIVE 2.0 guidelines were approved by the Ethical Committee for Animal Experimentation of JSC “Scientific Center for Anti-Infectious Drugs” (Approval No. 18/2024).

Animals were observed at least twice daily by trained staff for clinical signs, behaviour, and body condition. Specific humane endpoints were predefined in the study protocols, including >20% body weight loss, severe dehydration, persistent recumbency, loss of grooming behaviour, ruffled fur, hunched posture, self-mutilation, impaired mobility, or signs of uncontrolled pain or distress, such as vocalisation or rapid breathing. If any of these criteria were met, animals were humanely euthanised by isoflurane overdose followed by cervical dislocation, in accordance with Directive 2010/63/EU.

Anesthesia and analgesia protocols were applied as needed for each procedure to minimize pain and distress. No unplanned mortalities occurred. Refinement measures consistent with 3Rs were implemented, including reducing handling stress, using the least invasive procedures feasible, and closely monitoring animal welfare throughout the study. Humane endpoints were defined in advance, and no mortalities were observed during the study.

The experimental design, animal numbers, and outcome measures were predefined for each test, including dermal irritation, skin sensitization, acute dermal toxicity, pharmacokinetics, thyroid hormone assessment, anti-granuloma evaluation, and burn wound healing. Animals were housed under standard conditions (22 ± 2 °C, 50–60% humidity, 12 h light/dark cycle) with free access to food, water and environmental enrichment.

Randomization was performed prior to group assignment, and investigators remained blinded to treatment throughout the outcome evaluation. No animals were excluded, and no missing data were recorded.

For the burn wound model, ketamine (10 mg/kg, i.p.) was administered daily for seven days post-injury to provide analgesia. Surgical procedures, including cotton pellet implantation and burn induction, were carried out under ketamine/xylazine or isoflurane anaesthesia. When humane endpoint criteria were met, euthanasia was performed immediately, within 30 minutes of detection.

The duration of the experiments was as follows: 3 days for acute dermal irritation, 1 day for skin sensitization, 14 days for acute dermal toxicity, 8 days for the anti-granuloma study, and 10 days for burn wound healing. A total of 3 rabbits (acute dermal irritation), 40 guinea pigs (skin sensitization), 20 rats (acute dermal toxicity), 12 rabbits (pharmacokinetics), 9 rabbits (thyroid axis assessment), 15 rats (anti-granuloma), and 15 mice (burn wound healing) were used.

Except for the acute dermal toxicity test, all animals used were males to minimize sex-related variability. The dermal toxicity test included both males and females (5 of each per group) in accordance with OECD guidelines. Group assignments are described within the specific sections for each assay. No adverse events were observed; specifically, there were no mortalities, no local reactions at the application site, and no behavioral abnormalities in any of the experimental groups. All animals were euthanized at the planned study endpoint or upon reaching predefined humane criteria.

#### 2.3.1 Acute dermal irritation test.

The acute dermal irritation test followed the OECD Guidelines for the Testing of Chemicals, Test No. 404: Acute Dermal Irritation/Corrosion [15]. The study was conducted on three animals, each serving as its own control (untreated contralateral site), which, according to the OECD guideline, represents the minimum number required while ensuring ethical use of animals.

Three healthy New Zealand adult male albino rabbits (12 weeks old, body weight 1.9–2.4 kg) with intact skin were used. The animals were randomly assigned into three groups: Group 1 received 0.9% NaCl as the vehicle (negative control), Group 2 received 8% formaldehyde as a standard irritant (positive control), and Group 3 received 0.5 mL of undiluted Novostron.

Twenty-four hours before testing, a 5 cm x 5 cm area on the dorsal trunk of each rabbit was shaved and disinfected. A dose of 0.5 mL was applied to an approximately 6.25 cm^2^ area of skin, covered with a sterile gauze patch, and secured with an occlusive dressing for 4 hours. At the end of the exposure period, the patch was removed, and the test site was gently rinsed with lukewarm water, dried, and evaluated for dermal reactions. Observations were conducted at 1, 24, 48 and 72 hours post-exposure.

As no dermal reactions were observed, a confirmatory test was conducted using two additional rabbits. The procedure was repeated a total of three times. Following the third exposure, without irritation or corrosive effects, all animals were monitored for an extended observation period of 14 days.

Skin reactions were scored using the OECD 404 grading scale (0–4), where 0 indicates no irritation, and 4 denotes severe dermal response [15]. For each rabbit, dermal response scores at 24, 48 and 72 hours were summed and divided by three to obtain a mean irritation score per time point. These values were then averaged across all animals to calculate the Primary Irritation Index (PII).

The choice of the Organization for Economic Cooperation and Development (OECD) test guidelines over the ISO 10993 series was based on their long-standing application in regulatory toxicology, their comprehensive protocols for evaluating systemic toxicity, sensitization, and irritation, as well as their alignment with local regulatory requirements for pharmaceutical safety assessments. The OECD framework offers validated, reproducible methods for preclinical safety studies in rodents and non-rodents, making it well-suited for the initial evaluation of the Novostron formulation.

#### 2.3.2 Skin sensitization test.

The skin sensitization test followed OECD Guidelines for the Testing of Chemicals, Test No. 406: Skin Sensitization [16].

Forty healthy adult male guinea pigs (8 weeks old, bodyweight 670g - 750g) were randomly assigned into three groups: Group 1 received 0.9% NaCl (negative, control; n = 10), Group 2 received 0.1% 2,4-dinitrochlorobenzene (CDNB) in ethanol (positive control; n = 10), and Group 3 received 0.5 mL of undiluted Novostron (n = 20).

The selected Novostron dose was based on prior dermal safety results from the acute dermal irritation study, in which no visible skin reactions were observed. As recommended in OECD Test No. 406, the applied volume (0.5 mL) was sufficient to uniformly cover the ~ 6 cm^2^ test site, allowing for direct comparison across groups.

Each animal was shaved on the left flank and topically treated with the assigned test material. Dermal responses were evaluated 24 hours post-application. The sensitization test was repeated in three independent experiments under identical conditions.

Skin reactions were graded on a semi-quantitative scale [17]: 0 = no reaction, 1 = mild redness, 2 = moderate redness, 3 = intense erythema and/or edema. The sensitization rate and percentage were calculated based on the number of animals that exhibited a positive dermal response. These values were used to classify the sensitizing potential of Novostron according to OECD criteria.

#### 2.3.3 Acute dermal toxicity test.

The acute dermal toxicity test was conducted according to OECD Guidelines for the Acute Dermal Toxicity – Fixed Dose Procedure [18]. Each dose level included 5 animals per sex, consistent with OECD recommendations, which provides adequate sensitivity for detecting systemic toxicity while maintaining ethical standards for minimizing animal use.

Twenty healthy Wistar albino rats (8 weeks old, 210–230 g, 10 males and 10 females) were randomly assigned to a control group and a Novostron-treated group (n = 10 per group; 5 males and 5 females each). The dorsal area (~10–15% of the body surface) was shaved 24 hours before dosing. Novostron was applied once at a dose of 2000 mg/kg to the exposed skin area on Day 1. This dose represents the recommended limit in OECD Guideline No. 402 for substances that are anticipated to have low acute toxicity. Its selection was further supported by the known safety of the formulation’s components (iodine, dextrin, PVA, and trace metal salts). This application volume was adjusted according to each animal’s body weight to ensure accurate dosing across the designed dermal area.

Following treatment, animals were housed individually and observed for 14 days. Daily assessments included clinical signs of toxicity, dermal irritation, behavioral changes, and mortality. Body weights were recorded weekly.

On Day 15, all animals were euthanized under deep isoflurane anesthesia. Blood samples were collected via retro-orbital sinus puncture into K_3_-EDTA tubes (for hematology) and serum separator tubes (for biochemical analysis). Samples were centrifuged at 3000 rpm for 10 minutes, and the serum was separated within one hour of collection.

The liver, kidneys, and brain were excised, weighed, and preserved in 10% neutral-buffered formalin. Tissues were processed using standard histological techniques and examined under a light microscope (Carl Zeiss, Germany).

#### 2.3.4 ICP-MS and pharmacokinetic study.

Twelve healthy adult male New Zealand albino rabbits (10-week-old, body weight 1.2–1.8 kg) with intact skin were used. Novostron was applied topically to a shaved area of 25.0 cm^2^ at volumes of 1, 4, and 8 mL, corresponding to concentrations of 42.6 mg/cm^2^, 170.4 mg/cm^2^, and 340.8 mg/cm^2^, respectively. Rabbits were randomly assigned to four groups (n = 3 per group): Group 1 received 0.9% NaCl as the vehicle control, and Groups 2–4 received Novostron at 42.6, 170.4, and 340.8 mg/cm^2^, respectively.

Blood samples were collected into K₃EDTA tubes and gently inverted to prevent hemolysis. Samples were allowed to stand at room temperature for 1 hour and then centrifuged for 20 minutes at 1500 rpm. Plasma was transferred using an automatic dispenser into labeled polypropylene microtubes. Afterwards, 0.4 mL of 25% tetramethylammonium hydroxide was added and mixed thoroughly. Samples were then incubated in a drying oven at ~90°C for 3 hours. After cooling, the solution was transferred to a 10 mL volumetric flask and brought to volume with deionized water. The resulting analyte in 1% aqueous tetramethylammonium hydroxide was analyzed by ICP-MS using an Agilent 7500 instrument (Agilent Technologies, USA).

The following pharmacokinetic parameters were determined: total area under the serum concentration–time curve (AUC_total_), maximum serum concentration (Cmax), elimination half-life (t₁/₂), systemic clearance (Cl), mean residence time (MRT), and volume of distribution (V_d_).

#### 2.3.5 Impact on the thyroid hormones.

Nine healthy adult male New Zealand albino rabbits (10 weeks old, body weight 1.2–1.8 kg) were used. They were randomly assigned into three groups (n = 3 per group), and received a single topical application of Novostron at doses of 500, 2500 and 4000 mg/kg. Blood samples were collected into vacutainer tubes and centrifuged at 2000 rpm for 15 minutes. Plasma was then transferred into microtubes for the determination of T_3_, T_4_ and TSH levels using ELISA kits on Sunrise Microplate Absorbance Reader (Tecan, Austria).

#### 2.3.6 Cotton-pellet-induced granuloma formation.

The anti-inflammatory effect of Novostron on granulomatous inflammation was evaluated using the cotton pellet-induced granuloma model, as described by D’Arcy et al. [19]. The group size was selected based on prior publications using this model and on pilot observations in our laboratory that indicated biologically meaningful treatment effects. This sample size strikes a balance between the ability to detect relevant differences and ethical considerations to minimise animal use.

Fifteen male Wistar albino rats (8 weeks old, body weight 210–230 g) were randomly assigned into three groups (n = 5 per group): Group 1 – (negative control) received 0.9% NaCl; Group 2 – (positive control) received dexamethasone at a dose of 2 mg/kg administered intramuscularly; and Group 3 – received Novostron applied topically.

On Day 0, before implantation, the axillary region of each rat was shaved and disinfected. Under anesthesia, sterile cotton pellets (autoclaved, standardized size) were surgically implanted into the subcutaneous tissue of the axillary area. The incisions were closed using sterile silk sutures, and the animals were housed individually post-surgery.

Novostron was applied topically once daily for seven consecutive days. Dexamethasone was administered intramuscularly on the same schedule. The negative control group received no anti-inflammatory treatment.

On day 8, all animals were euthanized under anesthesia. The formed granulation-fibrous tissue was carefully dissected, and sterile swabs were used to collect samples from the exposed surface. To assess bacterial growth, the swab samples were plated onto tryptic soy agar (Sigma Aldrich, Merck, Darmstadt, Germany), with each measurement performed in triplicate.

For the determination of dry granuloma weight, tissue surrounding the cotton pellet was excised, dried in a hot-air oven at 60 °C, and weighed. Blood samples were collected via cardiac puncture into K_3_-EDTA tubes and centrifuged at 2000 rpm for 15 minutes to separate plasma for hematological analysis (Zytopia Ltd., China). Plasma samples were used to determine cytokine levels using ELISA kits (Thermo Fisher Scientific, Waltham, MA, USA) according to the manufacturer’s instructions.

#### 2.3.7 Burn wound model.

The burn wound model was established to evaluate the wound-healing activity of Novostron. A sample size of five animals per group was selected based on prior studies employing similar burn wound models [20], providing adequate sensitivity to detect treatment-related differences in wound closure and histological repair while adhering to ethical principles aimed at minimizing animal use.

Eighteen male BALB/c mice (14 weeks old, body weight 22–27 g) were randomly assigned into three groups (n = 6 per group): Group 1 – received topical 0.9% NaCl (negative control); Group 2 – received topical Betadine (positive control); and Group 3 – received topical Novostron.

Fifteen male BALB/c mice (14 weeks old, body weight 22–27 g) were randomly assigned into three groups (n = 5 per group): Group 1 – received topical 0.9% NaCl (negative control); Group 2 – received topical 5% Betadine (positive control); and Group 3 – received topical Novostron.

Betadine was used as the reference comparator to evaluate the efficacy of Novostron. It was applied at 5% once daily, following established protocols for topical burn care in rodents [4,45].

A full-thickness burn was induced using a modified Sector method [21]. After shaving the dorsal surface, mice were anesthetized with ketamine (100 mg/kg) and xylazine (10 mg/kg). A heated metal cylinder (20 mm diameter, filled with water at 90°C) was applied to the skin for 10 seconds, creating a circular wound that covered approximately 8% of the total body surface area (TBSA) [21].

To confirm full-thickness burn induction, representative skin samples were collected at T₀ (2 hours post-injury) from one mouse per group and processed histologically. The remaining fifteen mice received their treatments once daily for 10 consecutive days.

Post-burn analgesia was administered via intraperitoneal injection of ketamine (10 mg/kg) once daily for 7 days, following IACUC guidelines for burns ≥8% TBSA. Mice were monitored daily for signs of pain or distress, and the institutional ethics committee approved all procedures.

Wound diameters were measured on Day 0 and on Days 3, 5, 7, and 10 after burn induction. Assuming a roughly circular wound shape, the wound area was calculated as


$$\text{Area}=\pi {\text{r}}^{\text{2}},$$ (5)

This allowed for the calculation of wound area (mm^2^) at each time point for all treatment groups (0.9% NaCl, betadine, and Novostron). Wound contraction speed and the contraction coefficient were determined to evaluate the healing dynamics in each treatment group (0.9% NaCl, betadine, and Novostron).

One Day 10 (T_final_), mice were anesthetized with isoflurane for retro-orbital blood collection. Serum was separated by centrifugation (2000 rpm for 15 minutes) and stored for biochemical analysis. Mice were then sacrificed by cervical dislocation, and skin tissue from the wound margins was excised and fixed in 10% neutral-buffered formalin for 48 hours. Samples were embedded in paraffin, sectioned, and stained with hematoxylin, eosin (H&E) and Masson’s trichrome.

Epidermal thickness was measured in H&E–stained sections as the perpendicular distance between the basal and the stratum corneum layers, excluding regions containing hair follicles or exhibiting re-epithelialization artifacts. Measurements were obtained at five evenly spaced sites per wound section, and the mean value was recorded.

Collagen content and density were evaluated on Masson’s trichrome–stained wound sections. Digital images were acquired under identical magnification (400×) and illumination settings using an Axio Scope microscope (Carl Zeiss, Germany).

Collagen area was quantified in ImageJ (NIH, USA) after applying the Color Deconvolution (Masson Trichrome) algorithm to isolate the collagen channel. Collagen-positive (blue) regions within the dermis were segmented using a uniform threshold determined by the Otsu method. The collagen area fraction was calculated as the percentage of collagen-positive pixels relative to the total dermal area. Five non-overlapping dermal regions were analyzed per section, and the mean value was used for each sample.

Collagen density was determined from the same collagen channel by measuring the mean gray value of each ROI, with optical density (OD) calculated as:


$$OD\;{=\text{log}}_{\text{1}\text{0}}\left(\frac{\text{2}\text{5}\text{5}}{Mean\;gray\;value}\right)$$ (6)

Then the intensity of collagen staining was calculated according to the following formula:


$$Collagen\;Density\;(\%)\;{=\text{log}}_{\text{1}\text{0}}\left(\frac{\text{2}\text{5}\text{5}}{Mean\;gray\;value}\right)\text{x}\;\text{1}\text{0}\text{0}$$ (7)

This semi-quantitative parameter reflects the optical intensity of the collagen fibers and complements the collagen-positive area measurements obtained from the same histological sections. For each group, five random fields from five animals were analyzed under a light microscope (20 × objective, 10 × ocular lens; total magnification 200×).

### 2.4 Statistical analysis

Dermal irritation was evaluated using the Draize scoring system, which assesses the severity of erythema and edema following topical application [22]. Quantitative data are presented as mean ± standard deviation (SD). Normality of data distribution was assessed using the Shapiro–Wilk test. Homogeneity of variances was evaluated using Levene’s test for parametric analyses.

Depending on the experiment, statistical significance was determined using one-way or two-way ANOVA, or repeated-measures ANOVA (RM-ANOVA) for time-course data. Sidak’s post hoc test was applied for the acute dermal toxicity assay, and Dunnett’s multiple comparison test was used for all remaining experiments. Mean differences (MD) with corresponding 95% confidence intervals (CI) were calculated to indicate effect sizes and the precision of estimates.

For time-course analyses, RM-ANOVA was applied. Sphericity was not explicitly tested due to small sample sizes; however, RM-ANOVA is considered robust under these conditions, and conservative post hoc corrections (Sidak or Dunnett) were used appropriately.

All analyses used GraphPad Prism version 6.0 (GraphPad Software Inc., California, USA). A p-value of <0.05 was considered statistically significant.

## 3 Results and discussion

### 3.1 Physicochemical properties and NMR spectrum

The physicochemical properties of the synthesized Novostron complex were evaluated to assess its structural integrity, formulation stability, and pharmaceutical suitability. These parameters are crucial for ensuring batch-to-batch reproducibility and confirming the successful incorporation of iodine species within the polymer matrix. The assessment included pH, viscosity, density, iodine and iodide content, visual appearance, and overall yield (Table 1).

**Table 1:** Physicochemical parameters and composition of Novostron.

No	Name of indicators	Results
1	Empirical formula	(C_6_H_10_O_5_)_49_ 2LiI_3_ Mg(I_3_)_2_
2	Molecular Weight (g/mol)	9500
3	pH	4.23 ± 0.01
4	Viscosity (mm^2^/s) at 20 °C	12.06 ± 0.57
5	Density (g/cm^3^)	1.065 ± 0.02
6	Color	Dark brown
7	Iodine content (%)	8.13 ± 0.02
8	Iodide (mg/mL)	11.87 ± 0.02
9	Yield (%)	95

Novostron exhibited physicochemical characteristics consistent with stable polyiodide coordination within a polysaccharide matrix. The constructed empirical formula (C₆H₁₀O₅)₄₉·2LiI₃·Mg(I₃)₂ suggests an approximate molecular weight of ~9,500 g/mol, with the dextrin backbone contributing ~7,900 g/mol and the coordinated polyiodide salts accounting for the remainder. Although this does not represent a discrete molecular species, it reflects the relative composition and interaction patterns of the complex.

The stoichiometric model aligns with the consistent mass ratios of iodine, dextrin, and metal salts used during synthesis and is similar to previously described carbohydrate-based iodine complexes, where polyiodide species are stabilized within polymer matrices for controlled release and improved antimicrobial activity [23]. The high yield (95%) indicates efficient incorporation of iodine under the selected synthesis conditions. The dark brown color, slightly acidic pH (4.23 ± 0.01), viscosity (12.06 ± 0.57 mm^2^/s), and density (1.065 ± 0.02 g/cm^3^) all reflect the physicochemical properties typical of polyiodide–polysaccharide systems [24].

**Fig 1:**
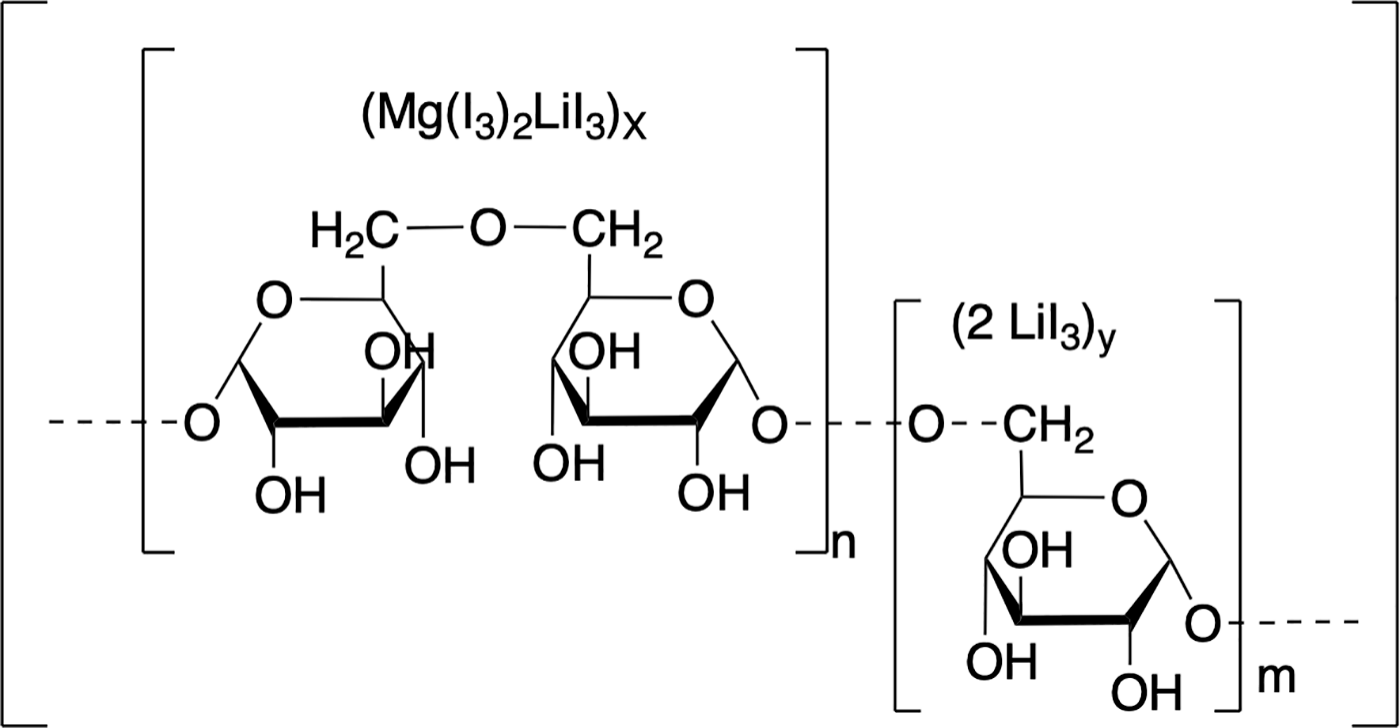
Schematic representation of the iodine-polymer complex structure (active center) in Novostron.

The active center of Novostron consists of a coordinated iodine–polymer network, stabilized primarily by dextrin and polyvinyl alcohol (PVA). This complexation enables the gradual release of iodine species, contributing to sustained antimicrobial activity and reduced cytotoxicity, which is commonly associated with free iodine. The synthetic route employed to obtain this system is protected under U.S. Patent No. 10,251,939 [2]. A schematic of the proposed active complex center is presented in Fig 1.

The structure depicts iodine predominantly in polyiodide form (I₃⁻), coordinated by lithium and magnesium cations and encapsulated within the hydrophobic cavities of dextrin chains. PVA contributes additional stabilization and impacts viscosity through intermolecular interactions. Iodine–polysaccharide complexes of this type have been extensively studied for their ability to modulate iodine release and enhance biocompatibility [25].

**Fig 2:**
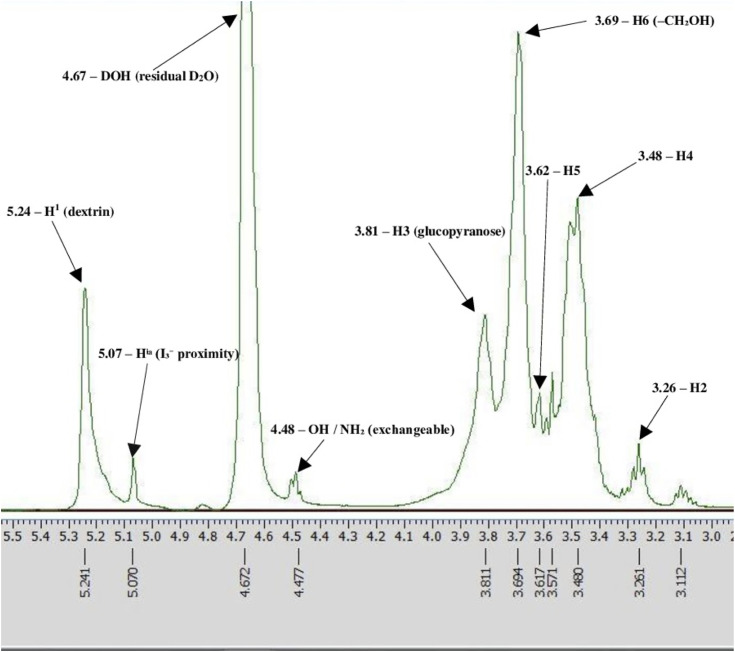
Representative ¹H NMR spectrum of Novostron in D₂O at 500 MHz. Characteristic chemical shifts are labeled: 5.24 ppm – H-1: anomeric proton of the α-glucopyranose unit in dextrin; 5.07 ppm – HO–I–I–I: triiodide-associated proton signal, reflecting polyiodide complexation; 4.67 ppm – DOH (residual HOD in D₂O): water exchangeable proton signal; 4.48 ppm – OH, SH, NH₂ (broad exchangeable protons): unresolved signals likely from hydroxyl or amine groups; 3.81 ppm – H-3; 3.69 ppm – H-6; 3.62 ppm – H-5; 3.48 ppm – H-4; 3.26 ppm – H-2.

The structural integrity of Novostron was further examined using ¹H NMR spectroscopy in D₂O (Fig 2).

The spectrum exhibits a complex but characteristic dextrin pattern. The downfield resonance at 5.24 ppm corresponds to the anomeric H-1 proton of α-linked glucopyranose units [26]. The signal at 5.07 ppm is assigned to proton environments associated with triiodide interaction (HO–I–I–I), supporting the presence of polyiodide species within the matrix [26].

Peaks between 3.2–3.8 ppm correspond to glucose ring protons (H-2 to H-6) and match reported spectra of dextrin and related polysaccharides [27]. The broad signal near 4.48 ppm reflects unresolved exchangeable protons, potentially from PVA or polysaccharide hydroxyl groups.

The absence of sharp peaks for low-molecular impurities and the presence of ordered peak clusters support the structural consistency of the formulation.

A summary of dextrin proton shifts of Novostron and component mixtures is presented in Table 2.

**Table 2:** ¹H NMR spectrum of Novostron. Chemical shifts (δ, ppm) of dextrin protons.

No.	Type of solution	Dextrin (ppm)							
		H1	P + C2	P + C3	P + C4	P + C5	P + C6	CH_2_	OH
1	Novostron	5.2	3.1	3.3	3.5	3.6	3.6	3.7	n/d
2	Dextrin	5.2	3.1	3.7	3.5	3.7	n/d	3.8	5.1
3	Dextrin + KI	5.2	3.1	3.3	3.3	3.5	3.7	3.8	5.1
4	Dextrin + I	5.2	3.1	3.3	3.5	3.7	n/d	3.8	5.1
5	Dextrin + I + KI	5.2	3.1	3.3	3.5	3.6	3.7	3.8	5.1
6	Dextrin + PVA + I + KI	5.3	3.1	3.3	3.5	3.6	3.7	3.8	5.1
7	Dextrin	5.2	3.1	3.3	3.5	3.6	3.7	3.8	5.1
8	Dextrin + Li + Ca + Mg	5.2	3.1	3.3	3.4	3.5	3.7	3.8	5.1
9	Dextrin + PVA	5.2	3.1	3.2	3.5	–	3.7	3.8	5.1
10	Dextrin + I_2_ + KI	5.2	3.1	3.2	3.6	3.6	3.7	3.8	5.1
11	Dextrin + PVA + I_2_ + KI	5.2	3.1	3.2	3.5	3.7	–	3.8	5.1
12	Dextrin + I_2_	5.2	3.1	3.2	3.5	3.6	3.7	3.8	5.1
13	Dextrin + KI	5.3	3.1	3.3	3.5	3.6	3.7	3.8	5.1
14	Dextrin + PVA + Li^+^ + Ca^2+^ + Mg^2+^ + KI + I_2_	5.2	3.1	3.3	3.5	3.6	3.7	3.8	5.1

**Fig 3:**
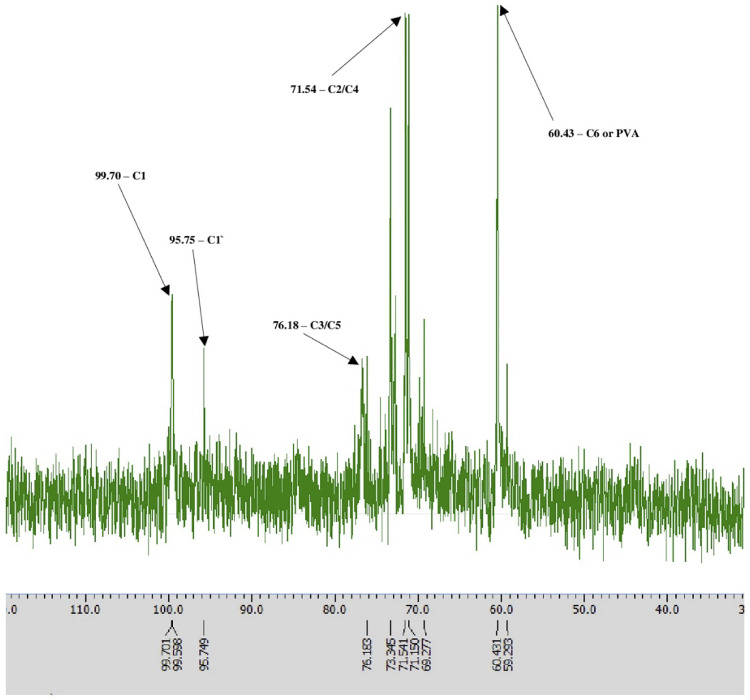
^13^C NMR spectrum of dextrin and PVA. ^13^C NMR spectrum of Novostron in D₂O. Major peaks were assigned based on known dextrin carbon positions and literature data: C1 at 99.70 ppm, modified C1′ at 95.75 ppm; C3/C5 at 76.18 ppm; C2/C4 at 71.54 ppm; C6 or PVA-associated carbons at 60.43 ppm.

To characterize the carbon backbone and verify the structural composition of the iodine–polymer complex, a ^13^C NMR spectrum of the Novostron formulation was recorded in D₂O solvent. The spectrum is presented in Fig 3.

The ^13^C NMR spectrum of Novostron displayed distinct resonances at δ 99.70, 95.75, 76.18, 71.54, and 60.43 ppm, consistent with the expected chemical shifts of carbon atoms in the glycosidic backbone of dextrin and the vinyl units of PVA. The signal at δ ~ 99.70 ppm is typically attributed to the anomeric C1 carbon of α-1,4-linked glucose units in dextrin, confirming its polysaccharide origin [27]. Peaks around δ 71.5–76.2 ppm are characteristic of C2–C5 ring carbons in the glucose monomers, while the resonance at δ ~ 60.43 ppm corresponds to the C6 primary alcohol group. The resonance at δ ~ 95.75 ppm may reflect minor structural rearrangements or the involvement of inter- or intramolecular hydrogen bonding, which are known to significantly influence the molecular architecture of polysaccharides, as demonstrated by systematic hydrogen-bond manipulation in related systems [28]. These signals support the use of dextrin and PVA in the final formulation and align with previous findings on iodine–polysaccharide supramolecular structures [28]. The absence of signals in the aromatic and carbonyl regions is consistent with the expected structure. It indicates no detectable contamination by aromatic or carbonyl-containing compounds, supporting the formulation’s compositional integrity.

The ^1^H NMR spectrum of Novostron confirms the presence of dextrin and PVA in their native forms, with distinct anomeric proton signals observed between 5.45 and 4.50 ppm, consistent with multiple glycosidic environments [28]. Notably, broad signals in the lower ppm range reflect the polymeric nature of dextrin and heterogeneity in chain length and branching typical of amylose–amylopectin mixtures. The ^13^C NMR spectrum further supports this composition, displaying well-resolved peaks at ~99.7, 95.7, 76.2, 71.5, and 60.4 ppm, characteristic of C1 (anomeric), C4, C3, C2/C5, and C6 positions of glucopyranose units, respectively [28]. The stability of these chemical shifts suggests that the glucan backbone undergoes minimal alteration during formulation.

In the presence of molecular iodine and potassium iodide, dextrin forms inclusion complexes wherein I₂ and polyiodides (I₃ ⁻ , I₅⁻) are stabilized within the V-type helical cavities of amylose. These complexes are responsible for the characteristic blue coloration and support a sustained release profile of iodine [29,30]. The role of PVA, whose C–H activation and possible syndiotactic ordering are detectable in ^1^H NMR, will likely involve additional stabilization via hydrogen or halogen bonding [30]. Collectively, the spectroscopic data validate the complex’s structure as a cardoxomer-type iodine delivery system with enhanced physicochemical stability and prolonged antimicrobial activity.

While the empirical formula and approximate molecular weight of Novostron (~9500 g/mol) were estimated based on the stoichiometric input of reactants and supported by structural analysis via NMR spectroscopy, the molecular weight has not yet been independently validated by size-exclusion chromatography with multi-angle light scattering (SEC-MALS) or alternative methods. Future work will address this limitation to characterize the polymer-iodine complex more precisely.

We also present a summary of the chemical shifts (δ, ppm) for dextrin protons within the Novostron formulation and in the test systems of its individual components (Table 3).

**Table 3:** ¹H NMR spectrum of Novostron. Chemical shifts (δ, ppm) of dextrin protons.

No.	Type of solution	Dextrin (ppm)							
		H1	P + C2	P + C3	P + C4	P + C5	P + C6	CH_2_	OH
1	Novostron	5.2	3.1	3.3	3.5	3.6	3.6	3.7	n/d
2	Dextrin	5.2	3.1	3.7	3.5	3.7	n/d	3.8	5.1
3	Dextrin + KI	5.2	3.1	3.3	3.3	3.5	3.7	3.8	5.1
4	Dextrin + I	5.2	3.1	3.3	3.5	3.7	n/d	3.8	5.1
5	Dextrin + I + KI	5.2	3.1	3.3	3.5	3.6	3.7	3.8	5.1
6	Dextrin + PVA + I + KI	5.3	3.1	3.3	3.5	3.6	3.7	3.8	5.1
7	Dextrin	5.2	3.1	3.3	3.5	3.6	3.7	3.8	5.1
8	Dextrin + Li + Ca + Mg	5.2	3.1	3.3	3.4	3.5	3.7	3.8	5.1
9	Dextrin + PVA	5.2	3.1	3.2	3.5	–	3.7	3.8	5.1
10	Dextrin + I_2_ + KI	5.2	3.1	3.2	3.6	3.6	3.7	3.8	5.1
11	Dextrin + PVA + I_2_ + KI	5.2	3.1	3.2	3.5	3.7	–	3.8	5.1
12	Dextrin + I_2_	5.2	3.1	3.2	3.5	3.6	3.7	3.8	5.1
13	Dextrin + KI	5.3	3.1	3.3	3.5	3.6	3.7	3.8	5.1
14	Dextrin + PVA + Li^+^ + Ca^2+^ + Mg^2+^ + KI + I_2_	5.2	3.1	3.3	3.5	3.6	3.7	3.8	5.1

Analysis of the ¹H NMR spectra revealed that the proton attached to the C3 carbon of the glucose unit in dextrin exhibited a chemical shift change of approximately 0.4 ppm in Novostron samples, as well as in the test solutions containing dextrin with KI, I₂, and their combination, compared to pure dextrin. This suggests a modification of the local magnetic environment and a possible structural reorganization of the dextrin macromolecule under the influence of iodine-containing components. The proton at C5 showed a smaller shift of about 0.1 ppm relative to pure dextrin, reflecting the synergistic influence of I₂ and I⁻ on proton shielding and local electron density. Similarly, the C6 proton exhibited a minor shift (~0.1 ppm), likely due to interactions between hydroxymethyl groups and iodide complexes.

The ¹H NMR spectra of dextrin displayed line broadening, especially in the lower field region, which can be attributed to the polymeric nature of dextrin and the presence of both amylose (~20–30%) and amylopectin (~70–80%) fractions resulting from incomplete hydrolysis. Amylose is a linear polymer consisting of 300–3000 (or more) glucose residues linked via α(1 → 4) glycosidic bonds, while amylopectin has a similar structure but with additional α(1 → 6) branching points. The average number of branches in amylose molecules ranges from 4 to 6 per molecule, with branching typically occurring in early stages of synthesis [31].

Dextrins, being polysaccharides with a low degree of polymerization, are known to form blue complexes upon interaction with iodide and molecular iodine, due to the inclusion of iodine molecules into the hydrophobic interior of amylose helices [32]. The addition of triiodide anion (I₃⁻) to a dextrin solution results in a characteristic blue coloration, as molecular iodine becomes encapsulated within the amylose helix [33–35]. Since molecular iodine (I₂) is hydrophobic, it preferentially occupies the interior of the amylose helix, while other iodine species (I ⁻ , I₃ ⁻ , In⁻) assist its solubilization in water [36,37].

The ¹H NMR data also suggest the formation of the V-type amylose complex (specifically V₆), consisting of six glucose units and three water molecules per helical turn. This allomorph is known for its intense blue coloration and the inclusion of hydrophobic iodine molecules within the helix alongside iodide anions [38].

Given the polysaccharide nature of dextrin and the resulting overlap of NMR signals, spin-spin coupling constants (J, Hz) could not be reliably determined, as their extraction is not feasible. The observed chemical shifts correspond to glucopyranose protons (H1–H6, CH₂, and OH groups). The influence of iodine and potassium iodide on the signal positions was minimal, indicating the preservation of the dextrin structure within the complex.

Overall, the observed chemical shift changes and their stability across all test systems confirm that the polysaccharide backbone of dextrin remains intact and undergoes no chemical degradation during complex formation with iodine.

In addition, we provide the corresponding table of ^13^C NMR chemical shifts (δ, ppm) for dextrin carbons in the Novostron formulation and its component systems (Table 4). The ^13^C NMR spectra show characteristic signals of dextrin glucopyranose units in the δ 60–105 ppm range. Signals at δ 95–105 ppm correspond to C1 carbons involved in α(1 → 4)- and β(1 → 4)-glycosidic linkages. The presence of both α and β signals confirms the incomplete hydrolysis of dextrin and the coexistence of linear and branched fragments. Signals for C2–C6 carbons were observed between δ 69–77 ppm, consistent with polysaccharide structures featuring α-glycosidic bonds.

**Table 4:** ^13^C NMR spectrum of Novostron. Chemical shifts (δ, ppm) of dextrin protons.

No.	Type of solution	Dextrin (ppm)							
		C1	α-bond	β-bond	C2	C3	C4	C5	C6
1	Novostron	95-105	95.749	99.598 and 99. 701	69.277	71.15	71.542	73.345	76.183
2	Dextrin	90-105	95.794	99.561 and 91.854	69.334	71.576	73.379	76.602	dextrin is not fully hydrolyzed
3	Dextrin + KI	90-105	95.937	99.523 and 92.131	69.363	71.528	72.825	76.259	dextrin is not fully hydrolyzed
4	Dextrin + I	90-105	95.775	99.533 and 91.911	69.306	71.538	73.35	76.564	dextrin is not fully hydrolyzed
5	Dextrin + I + KI	90-105	95.784	99.599 and 91.931	69.325	71.566	73.369	76.669	dextrin is not fully hydrolyzed
6	Dextrin	5.244	3.102	3.253	3.472	3.61	3.668	3.796	–
7	Dextrin + Li + Ca + Mg	5.242	3.104	3.253	3.407	3.541	3.668	3.796	–

Minor signal shifts (≤ 0.05 ppm) upon the addition of iodine and potassium iodide indicate the absence of destructive alterations in the dextrin macromolecule and preservation of its conformation. Slight changes in the intensity of C4 and C5 peaks may reflect local electronic density variations near glycosidic bonds caused by I₂/I⁻ complexation. The absence of significant shifts in the C1–C6 region supports that the interaction between dextrin and iodine species is physical (inclusion-based) rather than chemical.

Collectively, the ^13^C NMR results are consistent with the ¹H NMR data and confirm that the structural integrity of dextrin is maintained in the Novostron formulation, with iodine species interacting through non-destructive physicochemical inclusion mechanisms.

### 3.2 Acute dermal irritation test

To evaluate the potential irritant effects of Novostron, an acute dermal irritation study was carried out on rabbits according to the OECD guideline 404. The application site was visually inspected at 24-, 48-, and 72-hours post-exposure for any signs of erythema/edema, which were scored accordingly. Results are presented in Table 5.

**Table 5:** Acute dermal irritation scores and primary irritation index (PII).

Group	Dermal reaction	Erythema			Edema		
Negative control	Time point	24	48	72	24	48	72
	Mean	0	0	0	0	0	0
	SD	0	0	0	0	0	
	PII	0					
Positive control	Time point	24	48	72	24	48	72
	Mean	7.66	6.96	5.66	3.66	3.66	2.96
	SD	0.06	0.05	0.03	0.07	0.06	0.03
	PII	10.18					
Novostron	Time point	24	48	72	24	48	72
	Mean	0	0	0	0	0	0
	SD	0	0	0	0	0	0
	PII	0					

Overall, the Draize scoring results (Table 5 and S6 File in S1 Text) showed that Novostron caused no erythema or edema at 24-, 48-, or 72-hours post-application, with all animals scoring 0 across all time points – identical to the negative control. The positive control group, in contrast, exhibited marked irritation with high mean scores and a Primary Irritation Index (PII) of 10.18, confirming its strong irritant response. In comparison, the PII of Novostron was 0.0, indicating it is non-irritant and well-tolerated upon dermal application. Statistical analysis supported these findings: two-way repeated-measures ANOVA showed a highly significant treatment effect (p < 0.0001), with Dunnett’s post hoc tests revealing intense erythema and edema responses only in the positive control (MD 1.48–3.83, p < 0.0001), while Novostron did not differ from the negative control at any observation point.

These findings are notable given iodine’s well-documented potential to cause skin irritation in its free or tincture form. Polymer-based iodine delivery systems, such as Novostron, are designed to stabilize iodine release and reduce dermal reactivity, and the present results suggest that this objective was achieved. According to OECD Guideline 404, a score of 0 indicates a substance is non-irritant, consistent with similar literature reports for modified iodine formulations with controlled release [15,39].

**Fig 4:**
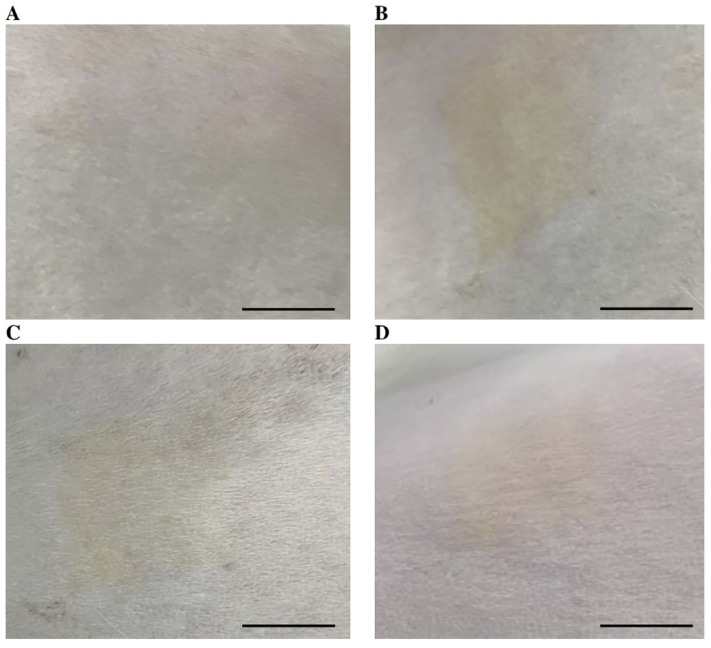
Skin condition at the test site of rabbits treated with Novostron: (A) Before application; (B) 24 hours post-application; (C) 48 hours post-application; (D) 72 hours post-application. Photograph courtesy of Tamari Gapurkhaeva. Copyright 2024.

In addition to numerical scoring, macroscopic assessment provides critical visual confirmation of skin response to test substances. Macroscopic photographs are presented in Fig 4.

Macroscopic examination of the treated sites visually confirmed the absence of dermal irritation in animals exposed to Novostron. As shown in Fig 4, the skin remained unchanged from baseline through 72 hours post-application, with no signs of erythema, edema, scaling, or surface disruption. This visual neutrality is crucial in the context of iodine-based compounds, which are often associated with localized irritation, discoloration, or dryness when used in traditional antiseptic formulations. The polymeric structure of Novostron likely facilitates a more controlled release of iodine, thereby minimizing acute inflammatory responses. These observations align with previous reports that iodine bound within polymers or surfactant complexes exhibits significantly lower irritation potential than free iodine [40]. The macroscopic findings and the irritation score data confirm that Novostron exhibit a favorable dermal tolerability profile.

### 3.3 Skin sensitization test

The skin sensitization potential of Novostron was assessed following OECD Test Guideline 406. No erythema or edema was observed in animals treated with Novostron or the negative control (0.9% NaCl) at 24-, 48-, or 72-hours post-challenge. In both groups, the sensitization rate was 0%, corresponding to Sensitization Class I (non-sensitizing or weak response). In contrast, all animals in the positive control group (treated with CDNB) exhibited pronounced erythema and edema, resulting in a 100% sensitization rate and classification as a Class V (extreme sensitizer). These findings are summarized in Table 6.

**Table 6:** Skin sensitization results and classification.

Group	Erythema	Edema	Sensitization Rate (%)	Sensitization Classification
Negative control (n = 10)	0	0	0 (0/10)	I – Weak
Positive control (n = 10)	1.40 ± 0.52	1.23 ± 0.44	100 (0/10)	V – Extreme
Novostron (n = 20)	0	0	0 (0/20)	I – Weak
**Group**			**Sensitization Rate (%)**	**Sensitization Classification**
Negative control			0	I – Weak
Positive control			100	V – Extreme
Novostron			0	I – Weak

Erythema/edema values are presented as Mean ± SD.

At 24 hours after the challenge application (Table 6 and S7 File in S1 Text), two-way repeated-measures ANOVA revealed a significant main effect of treatment for both erythema and edema (p < 0.0001), confirming distinct responses among groups, while interaction and repeat factors were negligible. Dunnett’s post hoc analysis showed that the positive control produced clear sensitization reactions, with pronounced erythema (MD = 1.40, 95% CI [1.33–1.47]) and edema (1.23, [1.12–1.35]; both p < 0.0001) compared with the negative control. In contrast, animals treated with Novostron exhibited no visible skin reactions. They did not differ significantly from the negative control, indicating the absence of sensitization or delayed hypersensitivity responses following dermal exposure.

In the skin sensitization assay, Novostron was classified as a non-sensitizer (Class I) based on the absence of reactions during the challenge phase (Table 6). This finding is notable, given that iodine-containing compounds, including well-established antiseptics such as povidone-iodine, are known to elicit sensitization responses in sensitive individuals occasionally [41]. The lack of any sensitizing response with Novostron may be attributable to its polymeric structure, which likely modulates iodine release and reduces dermal immune activation. Similar results have been reported for other iodine-containing complexes designed for controlled dermal delivery [42]. These results support the dermal safety of Novostron and justify its progression to acute dermal toxicity evaluation as a potentially well-tolerated iodine-based agent.

### 3.4 Acute dermal toxicity test

The acute dermal toxicity of Novostron was evaluated in rats over a 14-day observation period to assess systemic and local tolerability following topical exposure. No mortality, clinical signs of toxicity, behavioral abnormalities, or skin reactions were observed in either sex throughout the study. All animals demonstrated steady body weight gain over the two weeks, suggesting good systemic tolerance. Detailed results are presented in Table 7.

**Table 7:** Body weight of rats during acute dermal toxicity study.

Group	Sex	Body Weight (g)		
		Day 0	Day 7	Day 14
Control	Male	223.53 ± 8.26	229.32 ± 9.01	236.82 ± 9.78
	Female	219.91 ± 7.87	221.01 ± 7.86	225.82 ± 8.97
Novostron	Male	222.45 ± 8.16	229.75 ± 8.87	240.02 ± 8.77
	Female	219.57 ± 7.97	220.57 ± 8.15	226.22 ± 8.56

Data are presented as Mean ± SD, n=10 per group.

Body weight gain was observed in all experimental groups throughout the 14-day observation period (Table 7). Two-way ANOVA revealed that treatment had no significant effect on body weight at any time point (p > 0.05). In contrast, the sex factor was significant on both Day 7 (p = 0.0066) and Day 14 (p = 0.0025), reflecting the expected higher weights in males compared to females. No significant interaction between treatment and sex was detected. On Day 14, a small but statistically significant increase in mean body weight was noted in Novostron-treated males compared with control males (p < 0.05); however, the difference (≈3 g, < 2%) was within the range of normal biological variation and was therefore considered not treatment-related. Overall, all animals exhibited normal weight progression, indicating the absence of systemic toxicity following dermal exposure to Novostron.

These results confirm the absence of systemic toxicity, as sustained or increased body weight is a well-established indicator of physiological normalcy in subacute rodent toxicity studies. The non-significant interaction further suggests consistent tolerability of the compound across sexes, consistent with the expectations of the OECD 420 guideline for limit tests.

To explore the preliminary safety profile of Novostron, we evaluated its impact on the relative weights of the liver, kidney, and brain in male and female rats following acute dermal exposure. The results are presented in Table 8 and S8 File in S1 Text.

**Table 8:** Relative organ weights of rats following acute dermal exposure on Novostron.

Group	Sex	Relative weight of organs (mg organ weight/g body weight)		
		Liver	R. Kidney	Brain
Control	Male	31.15 ± 0.28	10.05 ± 0.21	6.78 ± 0.19
	Female	29.74 ± 0.11	8.47 ± 0.18	7.75 ± 0.16
Novostron	Male	32.27 ± 0.30**	10.24 ± 0.22	6.92 ± 0.18
	Female	30.05 ± 0.15	8.65 ± 0.19	7.81 ± 0.21

Data are presented as Mean ± SD, n = 10 per group; **p < 0.01 vs. control.

Analysis of relative organ weights revealed distinct patterns across tissues and sexes (Table 8). For the liver, both treatment (p = 0.0026) and sex (p < 0.0001) effects were significant, with males generally exhibiting higher relative liver weights and a slight but statistically significant increase in Novostron-treated males compared to controls. This indicates a sex-dependent hepatic response, with male rats showing greater sensitivity to treatment. Such hepatic hypertrophy is a typical, non-adverse adaptive response in rodent toxicology studies, often linked to enzyme induction rather than toxicity [43].

In contrast, the relative weight of the right kidney showed a strong influence of sex (p = 0.0001) but no significant treatment-related changes, indicating normal renal morphology following Novostron exposure. Similarly, a pronounced sex difference was observed in the brain (p = 0.0008), while neither treatment nor interaction effects were significant. The absence of interaction effects in the brain or kidneys supports a lack of systemic toxicity, and the observed liver changes fall within the known range of physiological adaptation [43].

To evaluate the systemic hematological safety of Novostron, a comprehensive analysis of hematological parameters was conducted in male and female rats following dermal application. The results are presented in Table 9.

**Table 9:** Hematological parameters of rats following acute dermal exposure to Novostron.

Name	Sex	Control	Novostron
RBCs, 10^12^/L	Male	5.72 ± 1.12	5.84 ± 1.09
	Female	5.57 ± 0.94	5.61 ± 1.05
HGB, g/L	Male	140.46 ± 12.13	139.67 ± 14.18
	Female	136.50 ± 15.39	134.33 ± 15.76
HCT, %	Male	39.74 ± 6.15	40.11 ± 7.08
	Female	35.85 ± 7.21	36.52 ± 6.98
MCV, fL	Male	74.07 ± 5.82	75.63 ± 6.01
	Female	72.59 ± 4.95	73.92 ± 4.89
MCH, pg	Male	24.25 ± 3.92	25.69 ± 4.02
	Female	23.01 ± 3.78	23.97 ± 3.91
MCHC, g/dL	Male	35.78 ± 6.62	37.00 ± 6.85
	Female	34.22 ± 5.97	35.14 ± 6.02
WBCs, 10^9^/L	Male	7.89 ± 0.88	8.15 ± 0.92
	Female	7.71 ± 0.80	7.85 ± 0.81
PLT,10^9^/L	Male	347.15 ± 61.92	355.59 ± 86.10
	Female	325.00 ± 74.33	328.24 ± 97.01

Data are presented as Mean ± SD, n = 10 per group; *p < 0.05, **p < 0.01 vs. control. Note: RBCs – red blood cells, HGB – hemoglobin, HCT – hematocrit, MCV – mean corpuscular volume, MCH – mean corpuscular hemoglobin, MCHC – mean corpuscular hemoglobin concentration, WBCs – white blood cells, PLT – platelets.

Analysis of hematological parameters revealed no major treatment-related effects of Novostron, although some sex-dependent differences were observed (Table 9 and S5 File in S2 Text). RBC counts were not significantly altered in males (MD = 0.1260, 95% CI: −0.1673 to 0.4193, P = 0.2040) or females (MD = 0.0440, 95% CI: −0.2493 to 0.3373, P = 0.6260). Hemoglobin (HGB) decreased significantly in females (MD = −2.172, 95% CI: −4.162 to −0.1821, P < 0.05) but not in males (MD = −0.7900, 95% CI: −2.780 to 1.200, P = 0.0623), while hematocrit (HCT) remained unchanged in both sexes (males: MD = 0.3680, 95% CI: −1.602 to 2.338, females: MD = 0.6680, 95% CI: −1.302 to 2.638, P > 0.05).

Mean corpuscular volume (MCV) increased significantly in males (MD = 1.560, 95% CI: 0.1910 to 2.929, P < 0.05) and showed a non-significant trend in females (MD = 1.336, 95% CI: −0.03297 to 2.705, P = 0.0422), while mean corpuscular hemoglobin (MCH) was increased in both males (MD = 1.440, 95% CI: 0.5535 to 2.327, P < 0.01) and females (MD = 0.9580, 95% CI: 0.07147 to 1.845, P < 0.05).

MCHC values were not significantly different between treatment groups (males: MD = 1.224, 95% CI: −1.731 to 4.179, females: MD = 0.9160, 95% CI: −2.039 to 3.871, P > 0.05), and WBC (males: MD = 0.2540, 95% CI: −0.04318 to 0.5512, females: MD = 0.1460, 95% CI: −0.1512 to 0.4432) and platelet counts (PLT; males: MD = 8.438, 95% CI: −17.38 to 34.26, females: MD = 3.240, 95% CI: −22.58 to 29.06, P > 0.05) were similarly unaffected.

Overall, these findings suggest that Novostron did not induce adverse hematological effects, and the observed variations primarily reflect normal sex-related differences possibly indicating adaptive or compensatory hematopoietic responses rather than pathological toxicity. Indeed, in hematology practice, small increases in MCV or MCH are typically interpreted in the context of erythropoietic stress or adaptation rather than toxicity per se [44].

A comprehensive biochemical panel was conducted to assess the potential systemic toxicity of Novostron and evaluate liver, kidney, and metabolic function in male and female rats following dermal administration. The results are presented in Table 10.

**Table 10:** Plasma biochemical parameters of rats following acute dermal exposure to Novostron.

Name	Sex	Control	Novostron
ALT, IU/L	Male	71.72 ± 8.78	72.58 ± 9.02
	Female	70.11 ± 7.99	71.58 ± 8.94
AST, U/L	Male	187.25 ± 11.52	188.00 ± 11.20
	Female	185.42 ± 10.98	186.95 ± 11.23
Scr, mg/dL	Male	0.47 ± 0.02	0.50 ± 0.02**
	Female	0.47 ± 0.01	0.49 ± 0.02**
BUN, mg/dL	Male	26.35 ± 3.91	27.85 ± 3.87
	Female	25.74 ± 3.12	26.91 ± 3.99
TG, mg/dL	Male	21.24 ± 3.22	22.62 ± 3.51
	Female	20.11 ± 3.41	21.79 ± 3.49
Total cholesterol, mg/dL	Male	40.23 ± 7.74	41.27 ± 8.01
	Female	39.82 ± 6.98	40.24 ± 7.05
LDL, mg/dL	Male	11.98 ± 2.75	12.52 ± 2.98
	Female	10.85 ± 2.22	11.68 ± 2.69
HDL, mg/dL	Male	22.07 ± 3.57	23.15 ± 3.98
	Female	21.58 ± 3.23	22.74 ± 3.87
Glucose, mg/dL	Male	104.35 ± 12.5	105.74 ± 13.10
	Female	103.75 ± 11.12	104.79 ± 11.82

Data are presented as Mean ± SD, n = 10 per group; *p < 0.05 vs. control: **p < 0.01 vs. control. Note: ALT – alaninaminotransferase, AST – aspartataminotransferase, Scr – serum creatinine, BUN – blood urea nitrogen, TG – triglycerides, LDL – low-density lipoprotein, HDL – high-density lipoprotein.

Plasma biochemistry analysis showed minor treatment- and sex-specific effects (Table 10 and S9 File in S1 Text). Alaninaminotransferase (ALT) was slightly higher in females following Novostron treatment (MD = 1.464 U/L, 95% CI: 0.183–2.745, p < 0.05) but not in males (MD = 0.856 U/L, 95% CI: −0.425–2.137, p > 0.05). Aspartataminotransferase (AST) remained unchanged in both sexes (male: MD = 0.748 U/L, 95% CI: −1.478–2.974, p > 0.05; female: MD = 1.536 U/L, 95% CI: −0.690–3.762, p > 0.05). Serum creatinine (Scr) increased slightly in males (MD = 0.034 mg/dL, 95% CI: 0.00529–0.0627, p < 0.05) but not in females (MD = 0.020 mg/dL, 95% CI: −0.00871–0.0487, p > 0.05), while blood urea nitrogen (BUN) was not significantly altered (male: MD = 1.500 mg/dL, 95% CI: −0.221–3.221, p > 0.05; female: MD = 1.164 mg/dL, 95% CI: −0.557–2.885, p > 0.05).

Lipid parameters showed modest changes: triglycerides (TG) increased in males (MD = 1.378 mmol/L, 95% CI: 0.044–2.712, p < 0.05) and females (MD = 1.680 mmol/L, 95% CI: 0.346–3.014, p < 0.05), LDL increased in females (MD = 0.774 mmol/L, 95% CI: 0.092–1.456, p < 0.05) but not in males (MD = 0.536 mmol/L, 95% CI: −0.146–1.218, p > 0.05), whereas HDL and total cholesterol were unchanged (HDL male: MD = 1.078 mmol/L, 95% CI: −0.361–2.517, p > 0.05; female: MD = 0.974 mmol/L, 95% CI: −0.465–2.413, p > 0.05; total cholesterol male: MD = 1.038 mmol/L, 95% CI: −2.960–5.036, p > 0.05; female: MD = 0.414 mmol/L, 95% CI: −3.584–4.412, p > 0.05).

Glucose was slightly affected by treatment overall (two-way RM ANOVA, F(1,4) = 7.779, p = 0.0494), however pairwise comparisons between Novostron and control were not significant in either males (MD = 1.398 mg/dL, 95% CI: −1.218–4.014, p > 0.05) or females (MD = 0.902 mg/dL, 95% CI: −1.714–3.518, p > 0.05). Overall, Novostron induced modest, predominantly non-adverse alterations in hepatic, renal, and lipid parameters, with minor sex-specific sensitivity, consistent with adaptive physiological responses rather than overt toxicity.

These findings align with previous reports on iodine-based formulations, exhibiting low systemic toxicity and high metabolic tolerance in animal models, further supporting the favorable safety profile of Novostron and reflecting normal lipid and carbohydrate metabolism under treatment [45].

**Fig 5:**
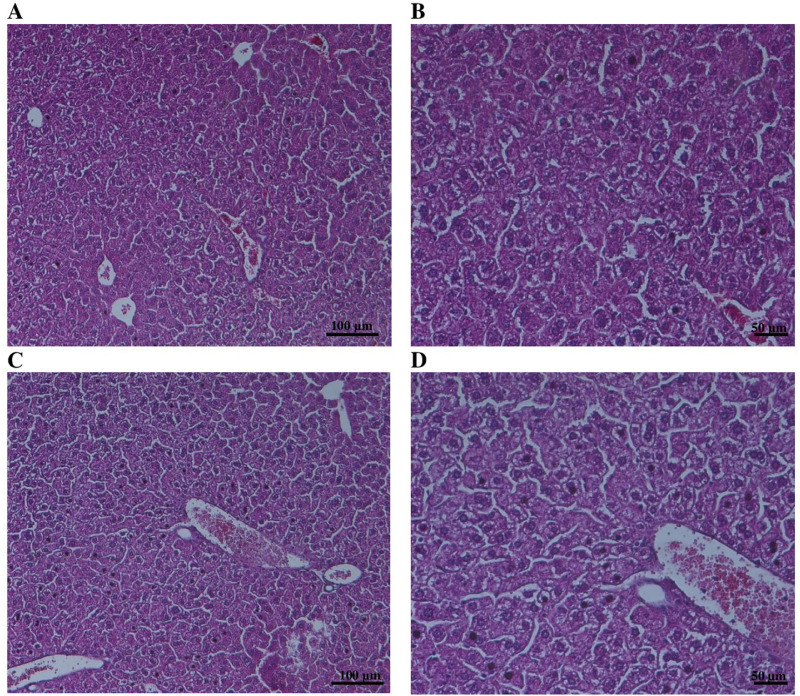
Representative images of H&E staining of liver: Control (A, B), Novostron (C, D). Images were captured with a 10 × objective and 10 × ocular lens (left panel) and a 20 × objective and 10 × ocular lens (right panel). Scale bars: 100 µm (left panel) and 50 µm (right panel).

**Fig 6:**
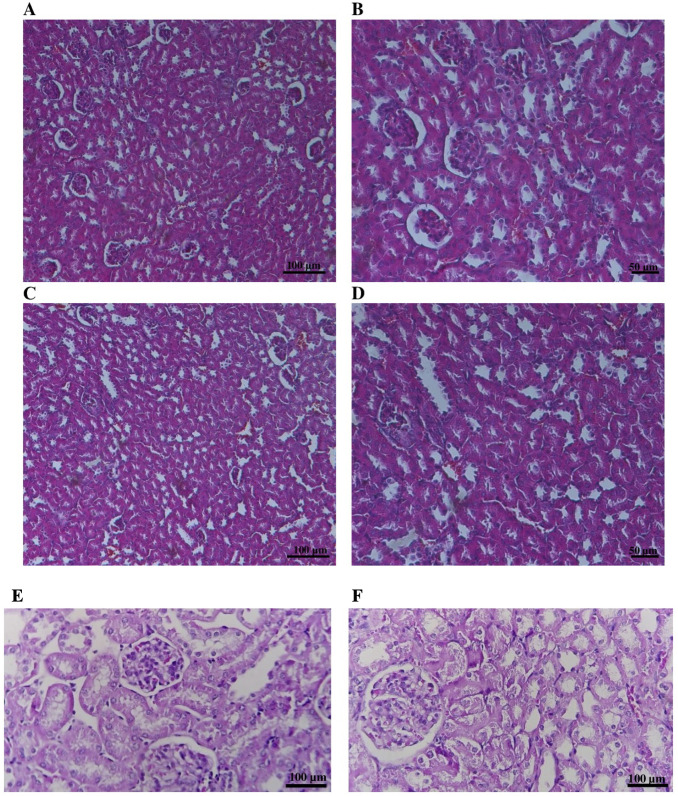
Representative H&E-stained kidney sections from control and Novostron-treated rats. Panels A–D show low- and mid-magnification images: Control (A, B) and Novostron (C, D), captured using a 10 × objective with 10 × ocular (A, C) and a 20 × objective with 10 × ocular (B, D). Scale bars: 100 µm (10×) and 50 µm (20×). Panels E and F show high-magnification images illustrating detailed renal histoarchitecture in Control (E) and Novostron (F) tissues (H&E stain, 400 × ; scale bar = 100 µm).

Histological examination of the liver and kidneys was performed to provide a detailed assessment of target organ responses. Representative microscopic images are shown in Figs 5 and 6.

Liver sections from Novostron-treated rats exhibited standard lobular architecture with well-organized hepatic cords, distinct sinusoidal spaces, and polygonal hepatocytes exhibiting uniform eosinophilic cytoplasm and centrally located nuclei. No cytoplasmic vacuolation, lipid accumulation or hydropic degeneration, corresponding to a vacuolation score of 0 on a semi-quantitative 5-point scale [46]. The preservation of standard hepatic architecture and the absence of treatment-related histopathological changes indicated a lack of hepatocellular stress or injury. These findings are consistent with morphologically healthy rodent liver parenchyma under physiological conditions, and suggest that there is no treatment-related hepatic toxicity [47].

Kidney histology revealed intact renal architecture in both groups, with well-preserved glomeruli and normal tubular morphology. Semi-quantitative scoring of renal injury parameters, including tubular vacuolization, epithelial degeneration, interstitial edema, and glomerular alterations, yielded a score of 0 for all parameters. There was no evidence of nephrotoxicity, such as tubular degeneration or interstitial inflammation, typically associated with cisplatin or gentamicin exposure [48–50]. The structural integrity of renal tissue supports the conclusion that the kidneys can tolerate Novostron at the tested dose.

### 3.5 Concentration of iodine in blood and pharmacokinetic parameters

**Fig 7:**
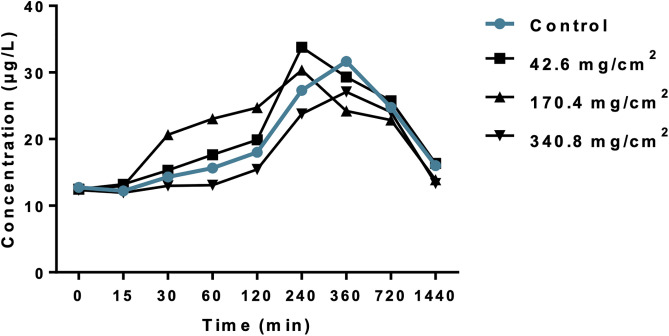
Concentration of iodine in blood of rabbits after topical treatment with Novostron at different timepoints: n = 3 per group.

Blood samples were collected at 0, 15, 30, 60, 120, 240, 360, 720, and 1440 minutes and analyzed using an ICP-MS system to determine the iodine concentration. The results are presented in Fig 7.

Repeated measures ANOVA revealed no overall significant effect of treatment on blood iodine concentration (F = 3.391, p = 0.076). Individual differences accounted for a significant portion of variance (F = 23.61, p < 0.0001). Dunnett’s post-hoc tests showed that only the 8 mL/25 cm^2^ dose resulted in a significant reduction in iodine levels compared to control (p < 0.01), whereas 1 and 4 mL/25 cm^2^ treatments did not differ significantly (Fig 7).

Key pharmacokinetic parameters were determined to describe the time course of iodine concentrations in blood after treatment. Results are presented in Table 11.

**Table 11:** Pharmacokinetic parameters of rabbits after topical treatment with Novostron.

Parameter	Result		
	42.6 mg/cm²	170.4 mg/cm²	340.8 mg/cm²
AUC_total_ (μg*h/L)	1082.80 ± 116.61	1091.87 ± 6.01	1001.33 ± 64.24
C_max_ (μg/L)	31.66 ± 6.90	33.77 ± 5.,70	30.35 ± 0.49
t_1/2_ (h)	21.80 ± 2.41	21.21 ± 2.83	22.51 ± 3.24
Cl (L/μg*h)	0.14 ± 0.01	0.27 ± 0.00	0.59 ± 0.04
MRT (h)	31.46 ± 3.47	30.61 ± 4.08	32.48 ± 4.67
V_d_ (L)	4.33 ± 0.45	8.32 ± 0.07	19.41 ± 3.97

Data are presented as Mean ± SD, n = 3 per group; *p < 0.05 vs. control.

Topical application of Novostron at 42.6, 170.4, and 340.8 mg/cm^2^ resulted in stable systemic exposure across most pharmacokinetic parameters (Table 11 and S4 File in S1 Text). Repeated-measures ANOVA showed no significant differences in AU_Ctotal_ (p = 0.444), C_max_ (p = 0.530), t_1/2_ (p = 0.702), or MRT (p = 0.702) among doses. In contrast, clearance (Cl) and volume of distribution (V_d_) increased significantly with dose (Cl: p = 0.0052; V_d_: p = 0.031), indicating enhanced systemic distribution at higher topical doses.

Post hoc Tukey’s tests confirmed significant differences for Cl and V_d_ between lower and higher doses, while AUC, C_max_, t_1/2_, and MRT remained unaffected. These data suggest that systemic exposure to Novostron is largely dose-independent for both peak and total plasma levels. However, higher topical doses lead to increased distribution and clearance, consistent with dose-dependent tissue absorption without disproportionate systemic accumulation.

This pattern aligns with previous reports showing that iodine applied topically may undergo variable dermal absorption and systemic clearance [51]. The absence of proportional increases in AUC or C_max_ implies that local retention and controlled release mechanisms may limit systemic accumulation, supporting the compound’s favorable tolerability in topical use.

### 3.6 Evaluation of thyroid hormones

**Fig 8:**
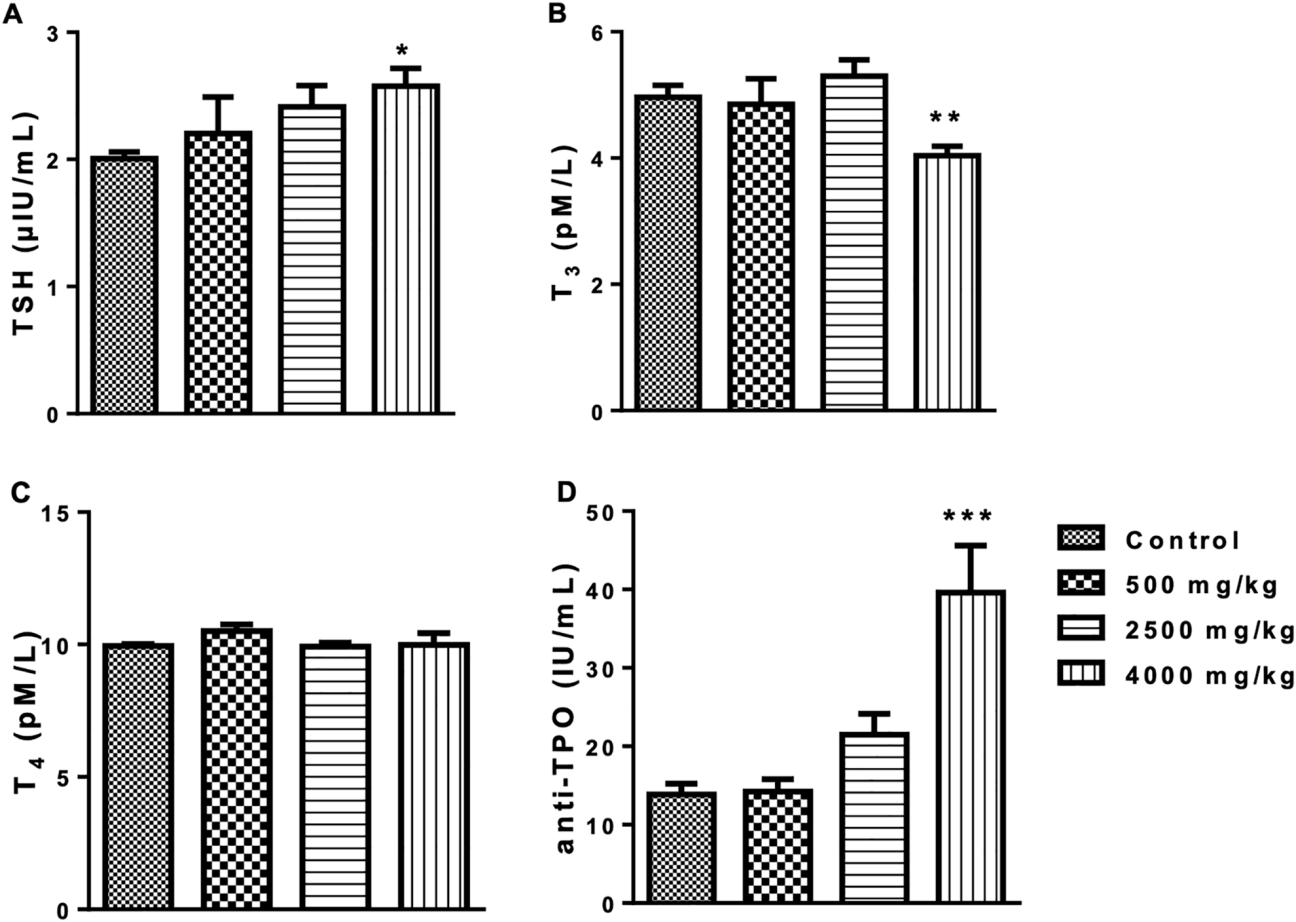
Serum levels of T₃, T₄, TSH, and anti-TPO antibodies: n = 3 per group, *p < 0.05, **p < 0.01 and ***p < 0.001 vs. control. Note: TSH – thyroid stimulating hormone, T_3_ – triiodothyronine; T_4_ – thyroxine and triiodo anti-TPO – anti-thyroid peroxidase.

The effects of Novostron on thyroid function were assessed in rabbits. Results are shown in Fig 8.

Analysis of thyroid function markers revealed dose-dependent changes following Novostron administration. Serum TSH levels showed a significant overall effect of treatment (F(3, 8) = 5.564, p = 0.0233), with a marked increase at 4000 mg/kg compared to control (p < 0.05). T₃ concentrations were also significantly affected (F(3, 8) = 11.77, p = 0.0026), with a pronounced decrease at 4000 mg/kg (p < 0.01). In contrast, T₄ levels did not differ significantly among groups (p = 0.0805).

A potent treatment effect was observed for anti-TPO antibodies (F(3, 8) = 36.68, p < 0.0001), where only the highest dose (4000 mg/kg) resulted in a significant elevation compared to the control (p < 0.0001). These findings suggest that high-dose Novostron alters thyroid regulatory balance, increasing TSH and anti-TPO while reducing T₃, whereas lower doses do not produce significant endocrine disruption. However, a histological examination of the thyroid gland was not performed; future studies are warranted to fully assess the potential endocrine effects of Novostron, particularly at higher doses.

### 3.7 Anti-granuloma activity

The anti-inflammatory efficacy of Novostron was evaluated in the cotton pellet-induced granuloma model in rats. Microbial contamination at the granuloma site was assessed on Day 8 to determine the compound’s antimicrobial activity, with each measurement performed in triplicate. The results of the bacterial burden analysis are presented in Table 12.

**Table 12:** Bacterial count at the granuloma site.

Bacterial count (10^3^ CFU/cm²)	
0.9% NaCl	7.51 ± 0.65
Dexamethasone	0.00 ± 0.00***
Novostron	0.00 ± 0.00***

Data are expressed as Mean ± SD (triplicate measurements), n = 5 per group; ***p < 0.001 vs. 0.9% NaCl.

Two-way repeated-measures ANOVA (Table 12) revealed a significant effect of treatment (F(2,12) = 668.6, p < 0.0001), while the interaction (F(4,24) = 0.1407, p = 0.9654) and repeat factor (F(2,24) = 0.1407, p = 0.8694) were not significant. Dunnett’s multiple comparisons test indicated that both dexamethasone and Novostron significantly reduced bacterial counts compared with 0.9% NaCl (MD = −751.7, 95% CI: −811.1 to −692.3, p < 0.0001 for both) (S2 File in S1 Text). These results support the antimicrobial activity of iodine-based formulations, which can reduce local bacterial burden without promoting resistance [52].

The comparable performance of Novostron and dexamethasone suggests that Novostron may deliver dual therapeutic benefits, including antimicrobial protection and inflammation control, potentially offering a non-corticosteroid alternative for localized treatment. This finding is consistent with prior reports on iodine-based agents that exhibit both antimicrobial and anti-inflammatory properties [53].

During the anti-granuloma study, body weight was monitored at three time points (Day 0, 3, and 8) to assess potential systemic effects of the treatment (Table 13).

**Table 13:** Body weight of rats during the anti-granuloma study.

	Day 0 (g)	Day 3 (g)	Day 8 (g)
0.9% NaCl	219.32 ± 7.54	220.96 ± 8.73	225.89 ± 8.31
Dexamethasone	219.73 ± 8.53	221.67 ± 9.24	226.31 ± 9.21
Novostron	219.61 ± 7.81	221.91 ± 8.49	225.86 ± 8.81
	Day 0	Day 3	Day 8
Group 1	219.32 ± 7.54	220.96 ± 8.73	225.89 ± 8.31
Group 2	219.73 ± 8.53	221.67 ± 9.24	226.31 ± 9.21
Group 3	219.61 ± 7.81	221.91 ± 8.49	225.86 ± 8.81

Data are expressed as Mean ± SD, n = 5 per group.

Two-way repeated-measures ANOVA (Table 13) revealed no significant interaction between treatment and day (F(4,24) = 0.64, p = 0.639) and no significant main effect of day (F(2,12) = 0.005, p = 0.995), indicating that body weights were consistent over time across all groups (S3 File in S1 Text).

Post-hoc Dunnett’s multiple comparisons test showed no significant differences in body weight between the 0.9% NaCl control group and either the dexamethasone (MD = 0.52 g, 95% CI [–12.96, 13.99]) or Novostron groups (MD = 0.40 g, 95% CI [–13.07, 13.88], not significant) at the measured time points. These results indicate that body weight remained comparable across all groups throughout the experiment. The absence of weight loss suggests good tolerability and minimal systemic toxicity, consistent with established criteria in preclinical models of inflammation [54].

The anti-inflammatory activity of Novostron was quantified by measuring the dry weight of excised granulomas, which allowed for separation into exudative, proliferative, and transudative components. This approach evaluates effects on both early (exudative) and late (proliferative) phases of inflammation. Percentage inhibition was calculated relative to the 0.9% NaCl-treated control (Table 14).

**Table 14:** Inhibition of Granuloma formation.

Group	Exudative weight (mg)	Proliferative weight (mg)	Transudative weight (mg)	Granuloma inhibition (%)
0.9% NaCl	411.49 ± 9.32	203.74 ± 9.05	207.75 ± 14.94	–
Dexamethasone	257.38 ± 25.87*	127.86 ± 32.26	129.52 ± 10.21*	37.65 ± 2.30
Novostron	286.8 ± 20.96*	128.36 ± 16.18	158.44 ± 9.09*	23.65 ± 2.28

Data are expressed as Mean ± SD, n = 5 per group; *p < 0.05 vs. 0.9% NaCl.

Both Novostron and dexamethasone significantly reduced the transudative weight of granulomatous tissue compared with the 0.9% NaCl control (F (2, 12) = 57.28, p < 0.0001). According to Dunnett’s post hoc test, the MD between the NaCl and dexamethasone groups was −78.24 mg (95% CI: −96.74 to −59.74, p < 0.001), while the MD between the NaCl and Novostron groups was −49.32 mg (95% CI: −67.81 to −30.82, p < 0.001).

Novostron produced a 23.65% inhibition of granuloma formation relative to NaCl, an effect lower than that of dexamethasone (37.39%) yet remarkable for a non-steroidal polymer–iodine complex. The response magnitude aligns with reported benchmarks for moderate efficacy in non-steroidal anti-inflammatory agents [55]. Mechanistically, the activity of Novostron may involve the modulation of fibroblast proliferation or the suppression of cytokine-driven granuloma maturation, processes central to chronic inflammation [56].

**Fig 9:**
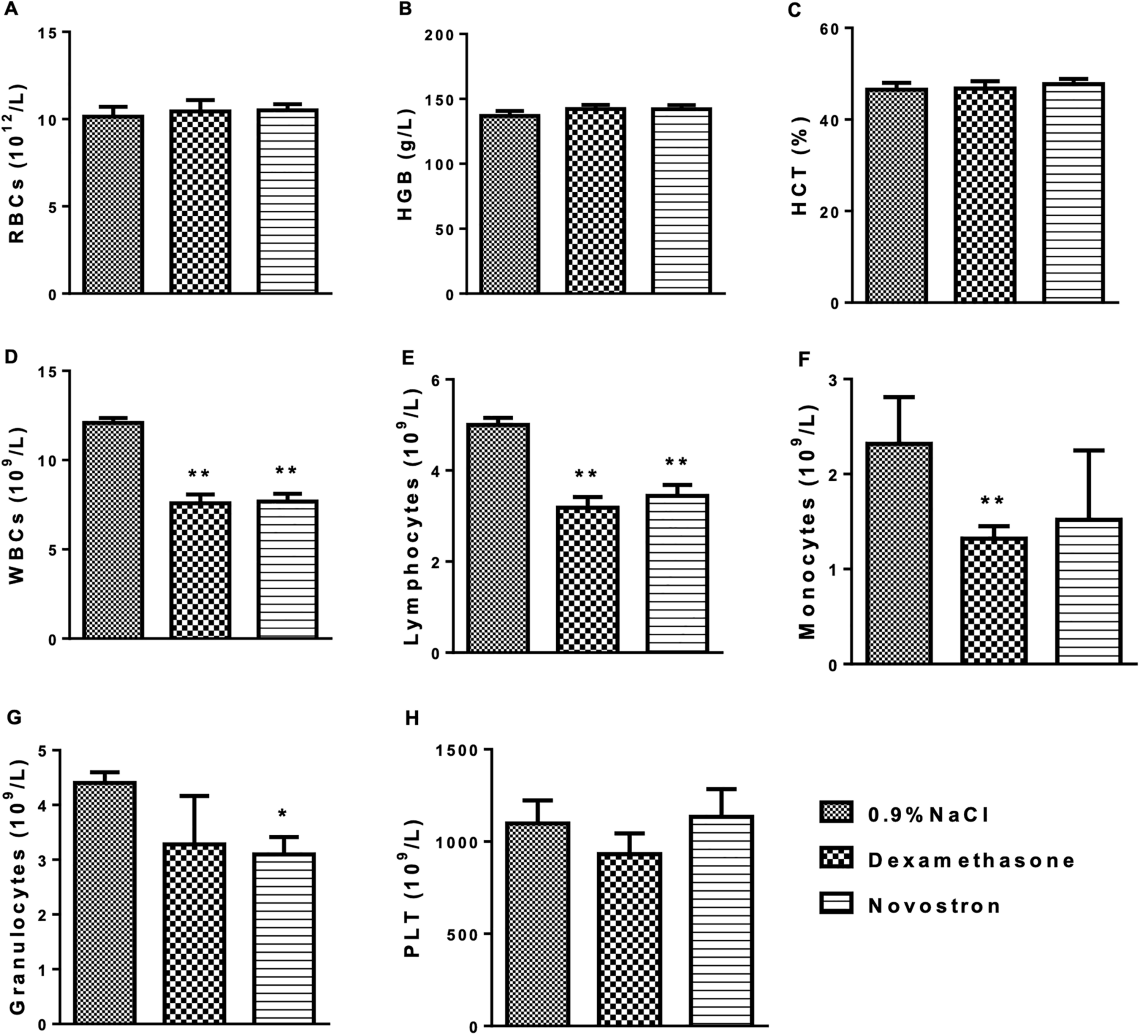
Hematological parameters of rats during Anti-Granuloma study: n = 5 per group, *p < 0.05 and **p < 0.01 vs. 0.9% NaCl. Note: RBCs – red blood cells, HGB – hemoglobin, HCT – hematocrit, WBCs – white blood cells, PLT – platelets.

In order to assess the hematological effects of Novostron, key blood parameters were analyzed in rats. The evaluation included red blood cell (RBC) count, hemoglobin (HGB), hematocrit (HCT), white blood cell (WBC) count, differential leukocyte counts (lymphocytes, monocytes, granulocytes), and platelet levels. These data are presented in Fig 9.

There were no significant differences in RBCs among the treatment groups (F (2, 12) = 0.6370, p = 0.5459). Dunnett’s multiple comparison test confirmed no significant difference between the NaCl and dexamethasone groups (MD = 0.3000; 95% CI: −0.5553 to 1.155) or between the NaCl and Novostron groups (MD = 0.3600; 95% CI: −0.4953 to 1.215; both p > 0.05) (Fig 9A).

HGB levels showed no statistically significant pairwise differences among groups, although the overall treatment effect reached significance (F (2, 12) = 4.03, p = 0.0458). Dunnett’s test indicated that neither dexamethasone (MD = 5.24 g/L; 95% CI: –0.07 to 10.55) nor Novostron (MD = 5.20 g/L; 95% CI: –0.11 to 10.51) differed significantly from NaCl (p > 0.05) (Fig 9B). HCT values also showed no significant differences (F (2, 12) = 0.96, p = 0.4121).

Dunnett’s multiple comparisons test revealed no significant differences between the NaCl and dexamethasone groups (MD = 0.26%; 95% CI: −1.99 to 2.51) or between the NaCl and Novostron groups (MD = 1.18%; 95% CI: –1.07 to 3.43; both p > 0.05) (Fig 9C).

A one-way ANOVA revealed a highly significant difference in WBCs among the experimental groups (F (2, 12) = 192.0, p < 0.0001). According to Dunnett’s multiple comparisons test, both the dexamethasone and Novostron groups showed significantly lower WBC levels compared with the NaCl group (MD = −4.50, 95% CI: −5.16 to −3.84, p < 0.0001; and MD = −4.40, 95% CI: −5.06 to −3.74, p < 0.0001, respectively). These findings indicate a pronounced anti-inflammatory effect of Novostron, comparable to that of dexamethasone (Fig 9D).

ANOVA analysis revealed a highly significant effect of treatment on lymphocyte counts (F (2, 12) = 103.8, p < 0.0001). Dunnett’s post hoc test confirmed that both dexamethasone and Novostron produced marked reductions in lymphocyte levels compared with the NaCl group (MD = −1.82, 95% CI: – 2.16 to −1.48, p < 0.0001; and MD = −1.56, 95% CI: −1.90 to −1.22, p < 0.0001, respectively) (Fig 9E).

A significant overall treatment effect on monocyte levels was observed (F (2, 12) = 5.31, p = 0.0223). According to Dunnett’s post hoc test, dexamethasone significantly reduced monocyte counts compared with the 0.9% NaCl group (MD = −1.00, 95% CI: −1.81 to −0.19, p < 0.05), whereas the Novostron-treated group did not show a statistically significant difference (MD = −0.80, 95% CI: −1.61 to 0.01, p > 0.05). This suggests that while Novostron exhibited a trend toward decreased monocyte levels, its effect was less pronounced than that of dexamethasone (Fig 9F).

A statistically significant effect of treatment on granulocyte levels was observed (F (2, 12) = 8.07, p = 0.0060). Dunnett’s multiple comparisons test revealed that both dexamethasone (MD = −1.12, 95% CI: −2.00 to −0.24, p < 0.05) and Novostron (MD = −1.30, 95% CI: −2.18 to – 0.42, p < 0.01) significantly reduced granulocyte counts compared with the 0.9% NaCl group (Fig 9G). No statistically significant differences were observed in platelet counts among the treatment groups (F (2, 12) = 3.46, p = 0.0651).

Although dexamethasone showed a decreasing tendency toward (MD = −166.0, 95% CI: –371.4 to 39.29) and Novostron showed a slight increase compared with the 0.9% NaCl group (MD = 36.4, 95% CI: −168.9 to 241.7), these changes did not reach statistical significance (Fig 9H). Overall, treatment with Novostron maintained stable erythrocyte indices while producing marked reductions in total leukocyte and lymphocyte counts, accompanied by moderate decreases in monocyte and granulocyte levels – effects comparable to those of dexamethasone.

**Fig 10:**
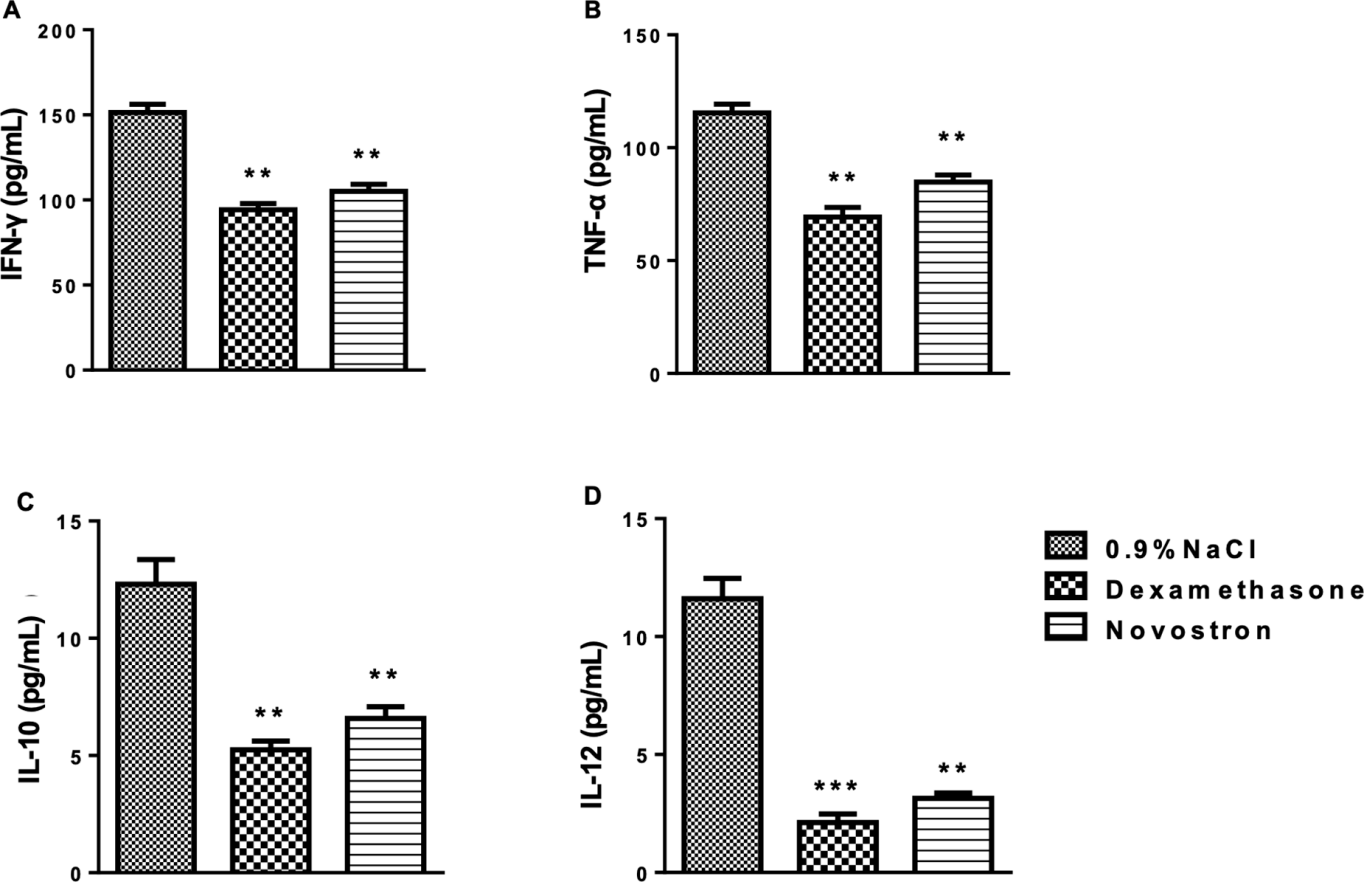
Cytokine profile of rats during anti-granuloma study (plasma): n = 5 per group, **p < 0.01, ***p < 0.001 vs. 0.9% NaCl.

To further characterize the anti-inflammatory profile of Novostron, we performed ELISA-based cytokine measurements on plasma samples, with results presented in Fig 10.

A one-way ANOVA revealed a highly significant treatment effect on IFN-γ levels (F(2,12) = 260.2, p < 0.0001). Both dexamethasone and Novostron produced a marked reduction in IFN-γ concentration compared to the 0.9% NaCl group (MD: −57.18 [95% CI: −63.84 to −50.52] and MD: −46.30 [95% CI: −52.96 to −39.64], respectively) (Fig 10A).

A significant treatment-dependent decrease in TNF-α levels was observed (F(2,12) = 191.1, p < 0.0001). Both dexamethasone and Novostron significantly reduced TNF-α concentrations compared with the 0.9% NaCl group (MD: −46.10 [95% CI: −52.11 to −40.09] and MD: −30.70 [95% CI: −36.71 to −24.69], respectively) (Fig 10B).

A highly significant treatment effect on IL-10 levels was detected (F(2,12) = 138.3, p < 0.0001). Both dexamethasone and Novostron markedly decreased IL-10 concentrations compared with the 0.9% NaCl group (MD = −7.06, 95% CI: −8.19 to −5.93 and MD = −5.72, 95% CI: −6.85 to −4.59, respectively) (Fig 10C).

A significant treatment-dependent modulation of IL-12 levels was observed (F(2,12) = 439.1, p < 0.0001). Both dexamethasone and Novostron produced a robust reduction in IL-12 compared with the 0.9% NaCl group (MD = −9.48, 95% CI: −10.36 to −8.60 and MD = −8.46, 95% CI: −9.34 to −7.58, respectively) (Fig 10D).

Analysis of cytokine profiles revealed that Novostron exerts a broad immunomodulatory effect comparable to dexamethasone. Treatment with Novostron significantly reduced the proinflammatory cytokines TNF-α and IFN-γ, both key mediators of macrophage activation and tissue injury in chronic inflammation.

Concurrently, Novostron enhanced IL-10 production—a central anti-inflammatory cytokine responsible for limiting immune-mediated tissue damage—while markedly suppressing IL-12, which drives Th1 differentiation and promotes sustained inflammation [57]. This cytokine balance shift toward an anti-inflammatory state supports the hypothesis that Novostron, as a polymeric iodophor, may exert corticosteroid-like regulatory effects on cytokine networks through non-steroidal mechanisms, potentially involving modulation of macrophage and T-cell signaling pathways [58].

These immunomodulatory effects are closely tied to the iodine release kinetics of the polymer matrix of Novostron, which enables sustained, low-level iodine release. This has been shown to inhibit NF-κB signaling and cytokine production in activated immune cells without causing cytotoxicity. This release profile may enable local immune modulation while minimizing systemic exposure, which explains the observed shifts in cytokine levels in both tissue and serum. Together, all findings of the anti-granuloma study highlight the potential of Novostron to reshape the local immune microenvironment within inflamed granulomatous tissue while promoting systemic resolution of inflammation.

### 3.8 Burn wound healing

**Fig 11:**
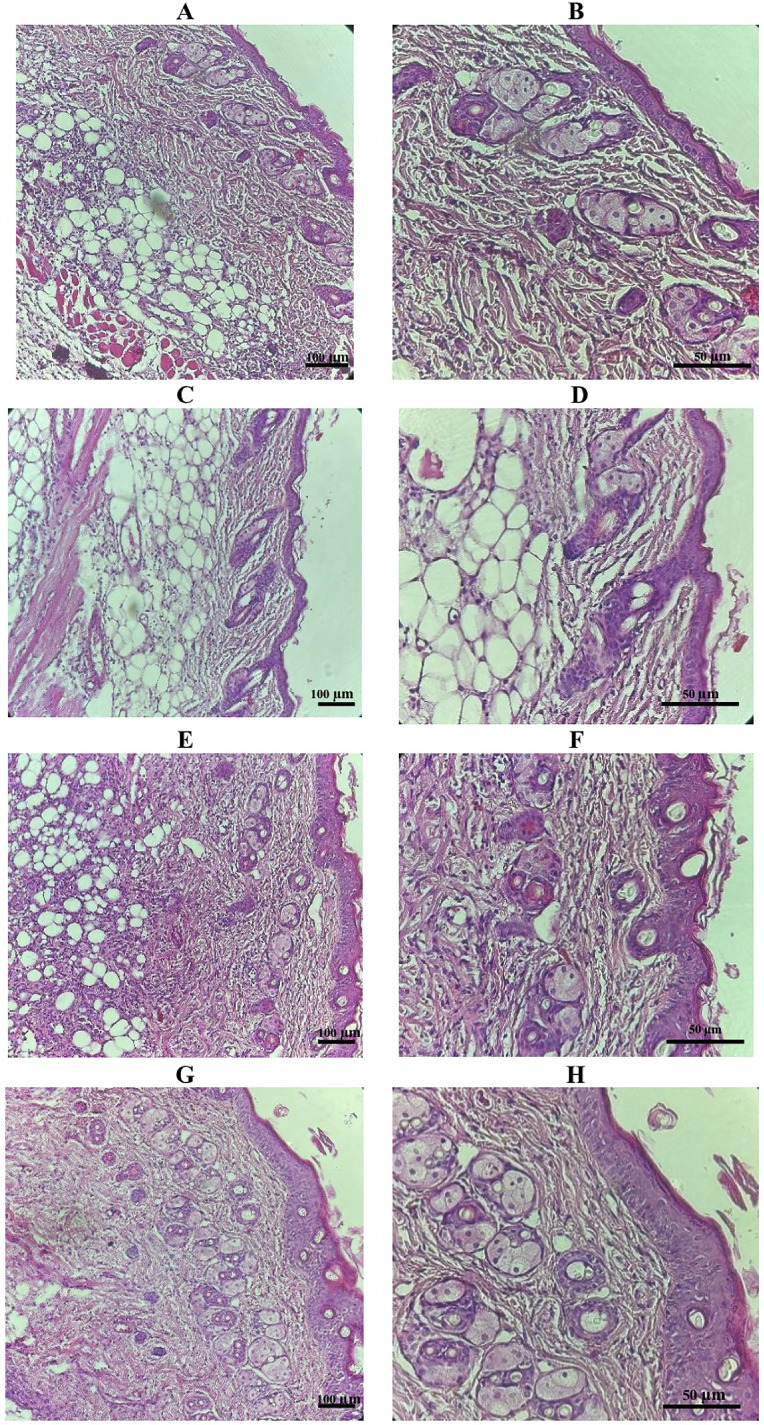
Representative images of H&E staining in the burn wound area: T0 (A, B), NaCl (C, D), betadine (E, F), Novostron (G, H). Images were captured with a 10 × objective and 10 × ocular lens (left panel) and a 20 × objective and 10 × ocular lens (right panel). Scale bars: 100 µm (left panel) and 50 µm (right panel).

**Fig 12:**
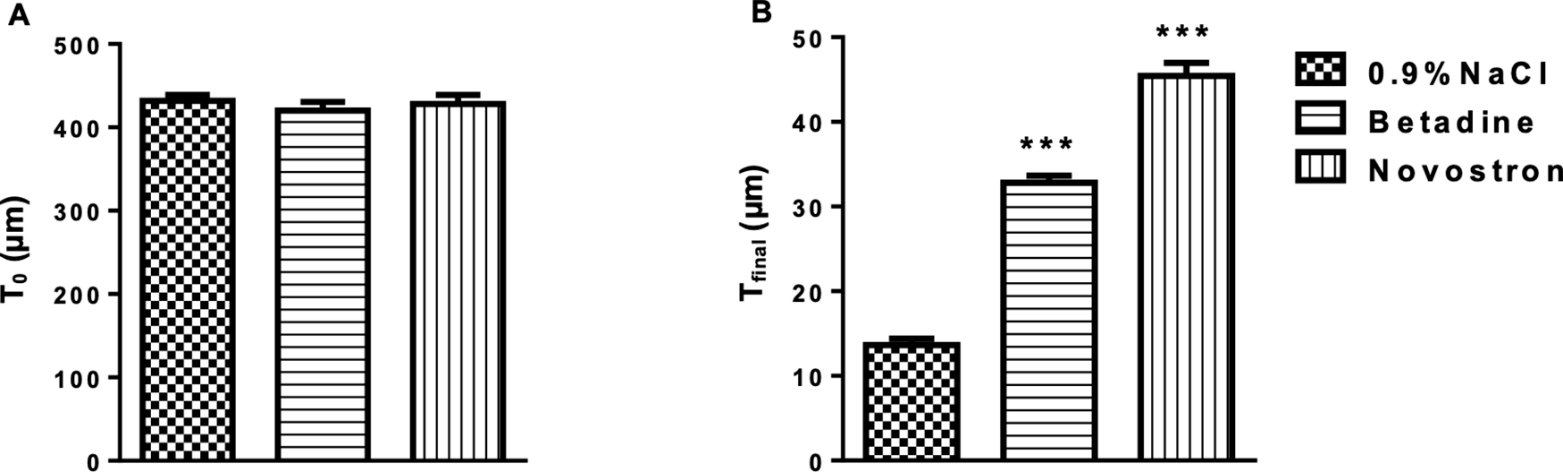
Quantitative histological assessment of burn wound healing: n = 5 per group, **p < 0.01, ***p < 0.001 vs. 0.9% NaCl.

Histological evaluation was performed at T₀ (2 hours post-burn) in representative animals (one animal per group, five fields per animal) to confirm consistent full-thickness burns across all groups before treatment. At T_final_, all animals were analyzed (five fields per animal). Representative microscopic photographs and quantitative assessments are presented in Figs 11 and 12, respectively.

Histological evaluation of H&E-stained skin sections provided insight into the degree of tissue repair and epidermal regeneration following burn injury and subsequent treatments. The tissue from the T_0_ group (Fig 11A) exhibited pronounced necrosis, widespread dermal disorganization, and the absence of intact epidermis, confirming the successful induction of full-thickness burns and validating the injury model [59].

In the NaCl-treated group, minimal epithelial recovery was observed, characterized by incomplete epidermal coverage and loosely arranged dermal collagen fibers (Fig 11B), which reflects impaired wound healing typically associated with unassisted repair [60].

The betadine-treated group (Fig 11C) exhibited partial re-epithelialization and improved dermal organization, consistent with the known antimicrobial and healing-promoting properties of povidone-iodine-based topical agents [61,62].

Notably, Novostron-treated tissue (Fig 11D) demonstrated the most substantial recovery, characterized by a continuous stratified epidermis, a prominent keratin layer, and dense, well-aligned collagen bundles in the dermis. These features indicate advanced wound remodeling. Similar histological outcomes have been reported for iodine-based treatments, supporting the hypothesis that Novostron enhance epithelial regeneration and collagen maturation, possibly through iodine-mediated immunomodulatory and pro-regenerative effects [63].

No significant differences in epidermal thickness were observed among treatments at T_0_ (F(2,12) = 1.953, p = 0.1844. Dunnett’s multiple comparisons revealed following: betadine vs. 0.9% NaCl, MD = –11.57, 95% CI –26.53 to 3.39; Novostron vs. 0.9% NaCl, MD = –3.71, 95% CI –18.67 to 11.25), confirming uniformity of the burn model (Fig 12A).

At T_final_, significant differences in epidermal thickness were observed among groups (F(2,12) = 1068, p < 0.0001). Both betadine and Novostron-treated wounds demonstrated significantly increased epidermal thickness compared with 0.9% NaCl (betadine, MD = 19.16, 95% CI 17.43–20.90; Novostron, MD = 31.76, 95% CI 30.03–33.49), indicating a strong wound-healing effect of Novostron (Fig 12B).

**Fig 13:**
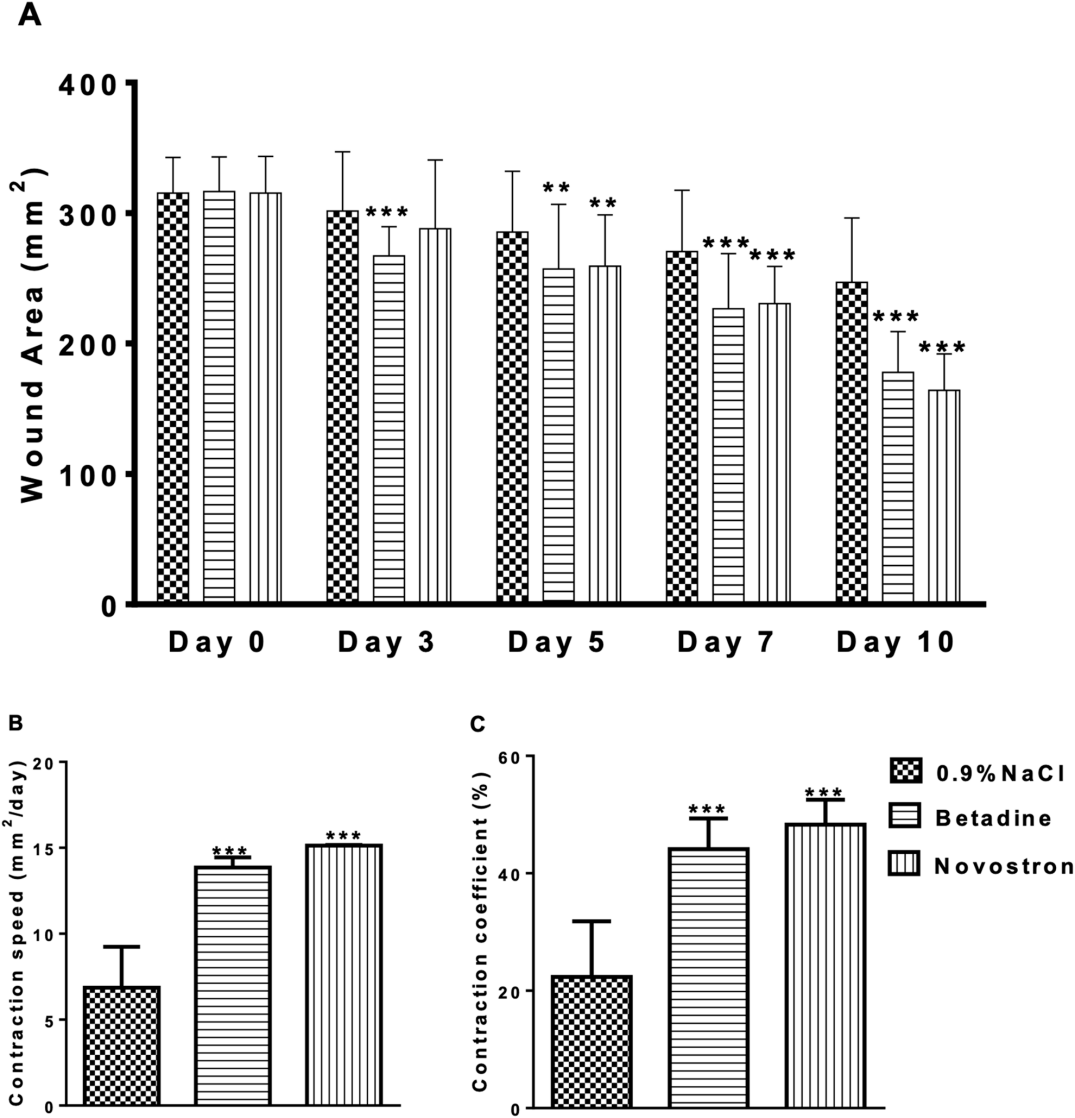
Burn wound contraction in mice during the burn wound healing study: n = 5 per group, **p < 0.01 and ***p < 0.001 vs. 0.9% NaCl.

In order to quantitatively evaluate the wound-healing efficacy of Novostron, the wound contraction rate was calculated throughout the 10-day wound-healing period. The results are presented in Fig 13.

Wound healing was evaluated by measuring wound area, contraction speed, and contraction coefficient across five time points (Days 0, 3, 5, 7, and 10) for all treatment groups (0.9% NaCl, betadine, and Novostron). A two-way repeated measures ANOVA of wound area revealed significant effects of time (p < 0.0001), treatment (p < 0.0001), and their interaction (p < 0.0001), indicating differential healing progression among groups.

Post hoc analysis revealed that betadine significantly reduced wound area compared to NaCl from Day 3 onward, while Novostron showed significant reductions from Day 5 onward. Both treatments achieved the most significant decrease by Day 10 (betadine −68.91 mm^2^; Novostron −82.73 mm^2^, p < 0.0001).

One-way ANOVA of wound contraction speed indicated significant differences among groups (F(2,12) = 49.30, p < 0.0001), with both betadine (MD = 7.00 mm^2^/day, p < 0.0001) and Novostron (8.28 mm^2^/day, p < 0.0001) demonstrating faster contraction than NaCl.

Similarly, the wound contraction coefficient differed significantly among groups (F(2,12) = 21.50, p = 0.0001), with betadine (21.80%, p < 0.001) and Novostron (25.95%, p < 0.001) showing enhanced closure relative to control.

Overall, these data indicate that both betadine and Novostron accelerated wound healing compared to NaCl, with Novostron showing a slightly greater effect at later stages of healing. The pronounced wound contraction observed in the Novostron group may be attributed to its combined antimicrobial and immunomodulatory properties, which have been reported to promote keratinocyte migration and granulation tissue formation [64]. Additionally, Bigliardi et al. [65,66] demonstrated that iodine complexes significantly accelerated wound closure in rats while reducing inflammatory infiltration, which supports the present findings for Novostron.

**Fig 14:**
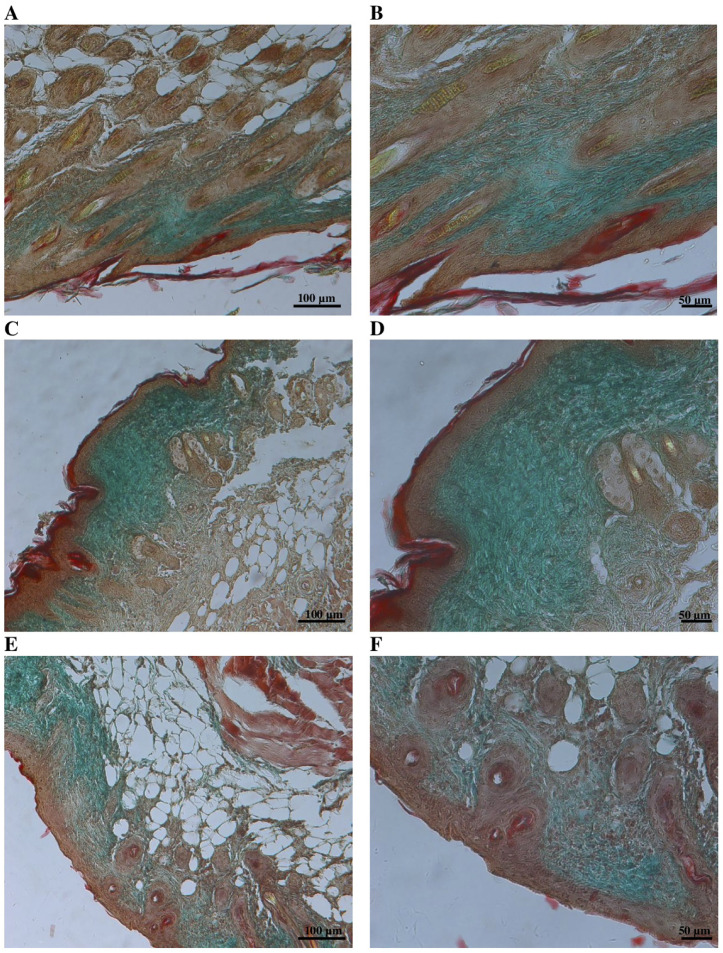
Representative images of Masson’s trichrome staining of the burn wound area: NaCl (A, B), betadine (C, D), Novostron (E.F). Images were captured with a 10 × objective and 10 × ocular lens (left panel) and a 20 × objective and 10 × ocular lens (right panel). Scale bars: 100 µm (left panel) and 50 µm (right panel).

Masson’s trichrome-stained wound sections were histologically evaluated to assess collagen deposition and dermal remodeling across treatment groups. Representative microscopic images are shown in **Fig 14**.

Masson’s trichrome staining revealed distinct differences in collagen distribution and organization between the treatment groups. In both betadine and Novostron-treated wounds, collagen deposition was evident, indicating active tissue repair (Fig 14). These patterns correspond with the known role of iodine-containing formulations in modulating inflammation and supporting balanced collagen synthesis during healing [67]. The organized collagen structure observed in the Novostron group suggests a favorable progression of tissue maturation, aligning with reports that structured collagen deposition contributes to improved functional recovery [68].

**Fig 15:**
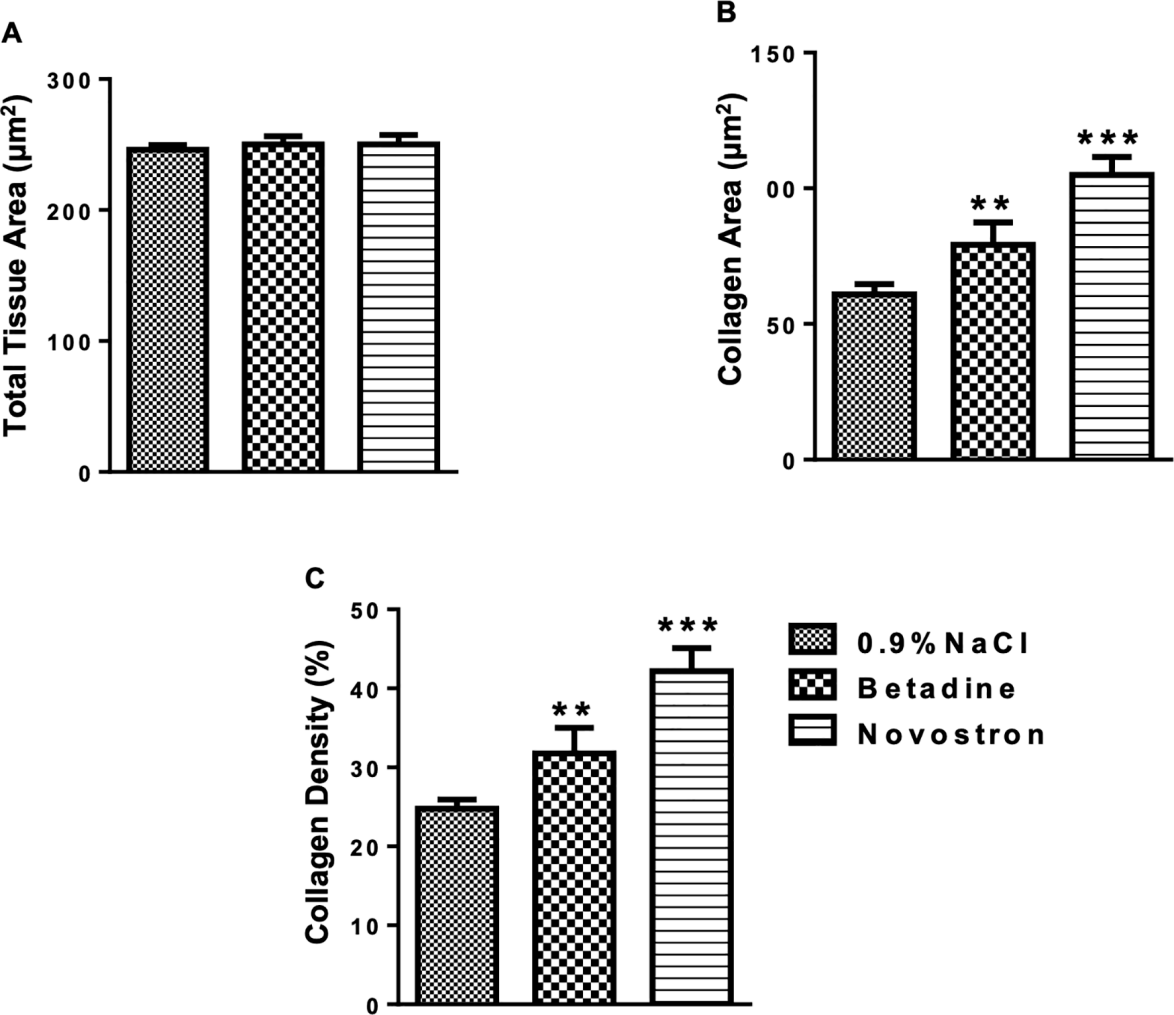
Quantitative analysis of collagen area and density in burn wounds of mice: **p < 0.01 and ***p < 0.001 vs. 0.9% NaCl.

Quantitative histological assessment was performed to evaluate the extent of dermal collagen deposition across treatment groups. Collagen area was measured as a percentage of total tissue area using Masson’s trichrome-stained sections and morphometric analysis. Results are presented in Fig 15.

Quantitative morphometric analysis demonstrated a significant increase in collagen deposition and density in the burn wound area following treatment with Novostron compared to the control and betadine group. One-way ANOVA for collagen area revealed a pronounced treatment effect (F(2,12) = 59.50, p < 0.0001, R^2^ = 0.9084), with Dunnett’s post-hoc test confirming that both betadine (MD = 18.25, p < 0.01) and Novostron (MD = 44.02, p < 0.0001) significantly increased the collagen-positive area relative to the 0.9% NaCl group.

Similarly, analysis of collagen density yielded consistent results (F(2,12) = 56.24, p < 0.0001, R^2^ = 0.9036), showing that Novostron treatment led to a markedly higher collagen optical density (MD = 17.41, p < 0.0001) compared to NaCl, while betadine produced a moderate but significant enhancement (MD = 7.02, p < 0.01).

The higher collagen density observed in the Novostron group may reflect a more advanced phase of extracellular matrix remodeling, potentially associated with increased fiber organization and early maturation of the collagen network [69]. However, this inference should be interpreted with caution, as the present analysis was based solely on optical density from MT staining and did not directly evaluate collagen types, alignment, or biochemical cross-linking. Additional histochemical and molecular assays would be necessary to validate whether the observed staining intensity corresponds to proper structural maturation.

To assess the systemic impact of burn injury and subsequent treatments, body weight was monitored throughout the 10-day post-burn period. The results are presented in Table 15 and S1 File in S1 Text.

**Table 15:** Body weight of mice during burn wound healing study.

Body Weight (g)				
	Day 0	Day 3	Day 7	Day 10
0.9% NaCl	24.87 ± 1.32	26.62 ± 2.00	28.25 ± 1.37	31.21 ± 1.30
Betadine	25.83 ± 1.52	27.40 ± 0.60	28.61 ± 0.73	29.83 ± 0.63
Novostron	25.53 ± 1.70	26.55 ± 2.17	28.23 ± 2.43	29.55 ± 2.06

Data are presented as Mean ± SD, n = 5 per group.

Throughout the burn wound healing study, body weights of mice remained stable across the experimental period, indicating the absence of systemic adverse effects related to treatment (Table 15). Two-way repeated-measures ANOVA showed significant effects of treatment (F (3, 36) = 285.2, p < 0.0001) and interaction (F (6, 36) = 8.228, p < 0.0001), but not of day (p = 0.8987). Dunnett’s multiple comparison test revealed no significant pairwise differences among treatment groups at any time point (p > 0.05).

The Novostron group exhibited stable weight gain comparable to betadine, supporting its non-inferiority in promoting post-burn recovery without impairing growth. These findings align with previous reports indicating that topical wound treatments, while affecting wound contraction and histological outcomes, often have minimal systemic effects on overall body weight unless infection or systemic inflammation is severe [70].

**Fig 16:**
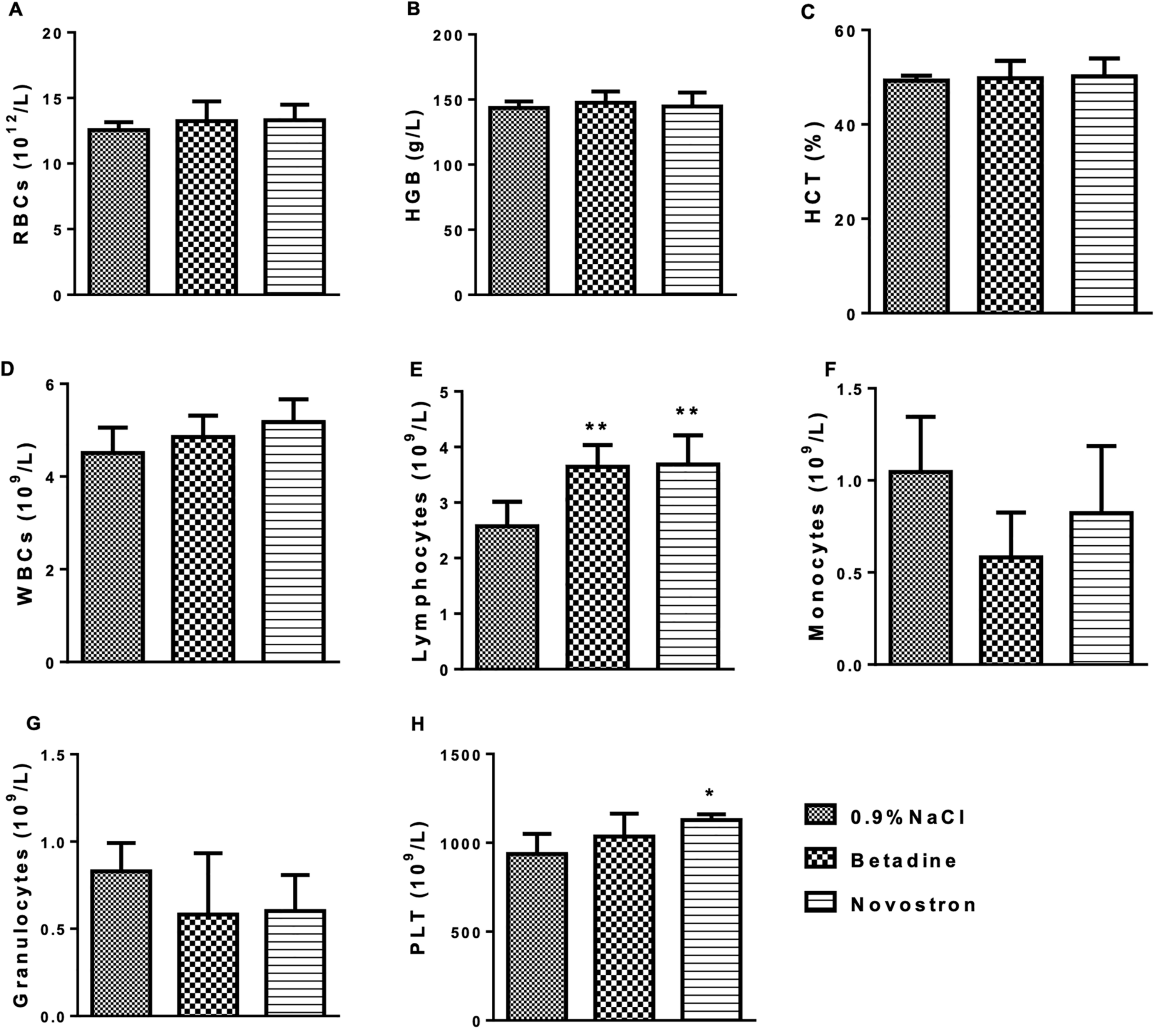
Hematological parameters of mice during burn wound healing study: n = 5 per group, *p < 0.05 and **p < 0.01 vs. 0.9%NaCl. Note: RBCs – red blood cells, HGB – hemoglobin, HCT – hematocrit, WBCs – white blood cells, PLT – platelets.

A one-way ANOVA was performed for each blood parameter to evaluate the treatment’s effects on hematological recovery in burn-injured mice. Results are presented in Fig 16.

Evaluation of peripheral blood parameters demonstrated that treatment with Novostron or Betadine did not significantly alter the levels of red blood cells (RBCs), hemoglobin (HGB), hematocrit (HCT), white blood cells (WBCs), monocytes, or granulocytes compared with the 0.9% NaCl control group (p > 0.05; one-way ANOVA).

In contrast, platelet (PLT) count showed a significant increase in the Novostron-treated group (p = 0.0351 vs. 0.9% NaCl; Dunnett’s test, p < 0.05), while betadine treatment did not cause notable changes. Additionally, a marked rise in lymphocyte count was observed in both betadine- and Novostron-treated groups (p < 0.01 vs. control).

These findings indicate that Novostron promotes moderate immune activation and mild thrombopoietic stimulation, reflected by elevated lymphocyte and platelet levels, while maintaining overall hematological stability during the burn wound healing process (Fig 16). The observed increases in lymphocytes and platelets following Novostron treatment suggest potential immunoregulatory activity and a capacity to enhance tissue repair, possibly via iodine-related modulation of oxidative stress and innate immune signaling.

**Fig 17:**
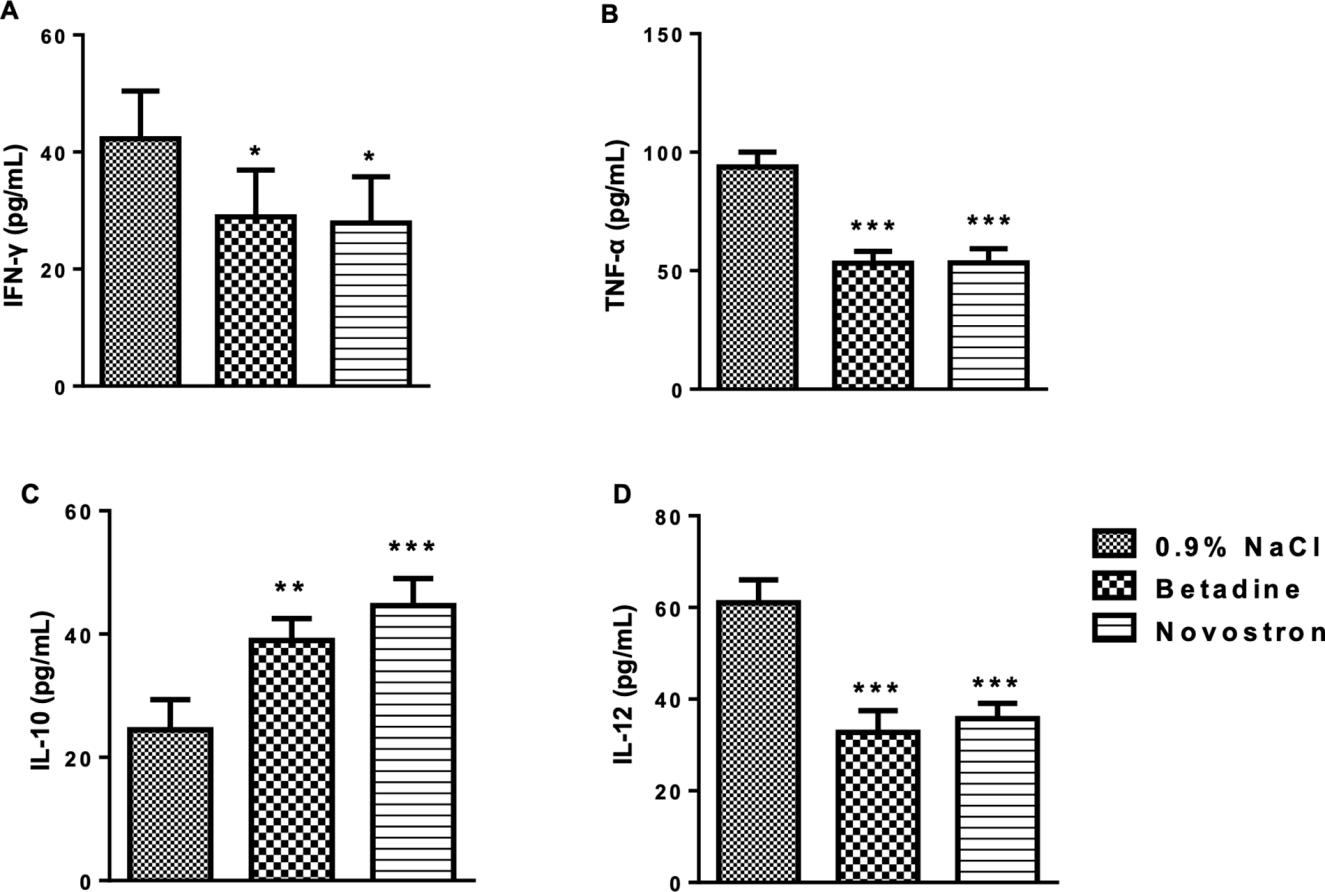
Cytokine profile of mice during burn wound healing study: n = 5 per group; *p < 0.05, **p < 0.01, ***p < 0.001 vs. 0.9% NaCl.

To further characterize the inflammatory response beyond hematological parameters, we next examined key cytokines involved in immune regulation during wound healing, including IFN-γ, TNF-α, IL-12, and IL-10. Results are presented in Fig 17.

Treatment with Novostron and betadine significantly modulated cytokine levels in the burn wound model. Both treatments reduced pro-inflammatory cytokines IFN-γ, TNF-α, and IL-12 compared with the NaCl control (IFN-γ: –14.4 and –13.3 pg/mL, TNF-α: –40.5 and –40.7 pg/mL, IL-12: –25.2 and –28.2 pg/mL for Novostron and betadine, respectively; all p < 0.05). Simultaneously, anti-inflammatory IL-10 was significantly elevated, with Novostron showing a more substantial effect (Novostron: + 20.2 pg/mL, betadine: + 14.5 pg/mL vs. NaCl; p < 0.001).

Our findings indicate that Novostron effectively suppresses Th1-mediated pro-inflammatory cytokines (IFN-γ, TNF-α, IL-12) while enhancing anti-inflammatory IL-10, producing a balanced immunomodulatory profile comparable to or slightly stronger than betadine. This pattern of cytokine modulation is consistent with previous reports, which show that topical interventions for burn wounds can influence both local and systemic immune responses, thereby supporting hematological stabilization during healing [70].

### 3.9 Limitations

While our study provides comprehensive preclinical data on the safety and wound-healing potential of Novostron, several limitations should be considered when interpreting the findings and their translational implications. These limitations primarily relate to study design, comparative formulation aspects, and the scope of physiological and histological assessments performed.

Given the iodine-based nature of Novostron, potential systemic absorption and effects on the thyroid axis warrant consideration. Although serum T₃, T₄, TSH, and anti-TPO levels were measured, histological analysis of the thyroid gland was not performed, limiting assessment of potential tissue-level effects. In addition, the study did not include measurements of dermal or urinary iodine levels. Nevertheless, the topical application was limited in dose and area, and no overt signs of systemic toxicity were observed in body weight or hematological parameters. Future GLP-compliant toxicology studies will include a comprehensive evaluation of systemic iodine exposure and thyroid function to inform safe dosing strategies and exposure matching relative to other iodine-based formulations.

A further limitation is the absence of a control group treated with the Novostron vehicle without iodine, which limits the ability to distinguish the contribution of the polymer matrix from that of iodine to the observed effects. However, previous data [56] support the overall efficacy of Novostron and help contextualize the antimicrobial and wound-healing outcomes, while acknowledging that the specific contribution of the vehicle remains to be determined in future studies.

In the acute dermal toxicity model, detailed morphological assessment of epidermal and dermal cells was not performed. Future investigations will include cellular-level characterization to complement histopathological findings and provide deeper mechanistic insights. Similarly, in the skin sensitization test, representative images were not obtained, as assessments were performed according to OECD TG 406 guidelines based on visual grading of erythema and edema reactions. Photographic documentation will be included in future studies to enhance transparency and reproducibility.

The use of the granuloma model represents another limitation, as it reflects localized inflammatory processes that may not fully correspond to systemic anti-inflammatory effects. While Novostron has been previously evaluated in an infected wound model with histological assessment [64], the present study focused on controlled tissue remodeling and immune cell infiltration. Given the known cytotoxicity of iodine-based compounds, some modulation of inflammation may also result from reduced immune cell viability rather than pure anti-inflammatory activity.

In the burn wound healing model, collagen organization and maturation were inferred indirectly from staining intensity, without complementary biochemical validation. Although histological analyses (H&E and Masson’s trichrome) were performed, macroscopic wound images were not captured. Furthermore, Novostron was not directly compared with povidone-iodine (betadine), an established iodophore. While both formulations were applied with the intention of matching iodine content, their differing physical forms (polymeric vs. aqueous) result in distinct release kinetics and local tissue exposure. Consequently, the observed differences in wound-healing outcomes should be interpreted with caution, as they may reflect formulation-specific pharmacodynamics and iodine bioavailability rather than strictly equivalent dosing. The study also used betadine at its standard clinical concentration for practical relevance; however, iodine equivalence between Novostron and betadine was not adjusted, which may limit direct dose-based comparisons and translational interpretation of the findings.

Finally, the study was conducted exclusively in preclinical animal models, including male New Zealand albino rabbits, male guinea pigs, male and female Wistar rats, and male BALB/c mice. These species may not fully replicate human skin physiology or wound-healing dynamics. Differences in skin thickness, barrier function, immune response, and iodine metabolism, as well as sex-related hormonal factors, may influence outcomes. Caution is therefore warranted when extrapolating these findings to humans, particularly to vulnerable populations such as pediatric patients or individuals with diabetic wounds, whose healing and immune responses differ significantly from those of healthy individuals. Controlled clinical trials will be necessary to confirm the safety and efficacy of this approach in diverse human populations.

## 4 Conclusion

This preclinical study demonstrates that Novostron, a novel polymer–iodine complex, has a favourable safety profile and therapeutic potential for topical wound management. NMR and physicochemical analyses confirmed the structural stability and controlled release of iodine. Pharmacokinetic evaluation showed stable systemic exposure across doses, with increased clearance and tissue distribution at higher concentrations but no disproportionate systemic accumulation. Thyroid hormone levels (T₃, T₄, TSH) remained within normal ranges, indicating preserved thyroid function.

Novostron exhibited no signs of dermal irritation, sensitization, or systemic toxicity, and significantly enhanced wound repair in granuloma and burn models through improved epidermal regeneration, collagen deposition, and reduced inflammation. These results suggest that controlled iodine release from the polymer matrix enables localized antiseptic action with minimal systemic exposure, supporting further clinical development of Novostron for wound-healing applications.

## Supporting information

S1 Text **S1 File.** Body weight of mice during burn wound healing study. **S2 File.** Bacterial count at the granuloma site. **S3 File.** Body weight of rats during the anti-granuloma study. **S4 File.** Pharmacokinetic parameters of rabbits. **S6 File.** Acute Dermal Irritation Scores and Primary Irritation Index (PII). **S7 File.** Skin Sensitization Results and Classification. **S8 File.** Relative organ weights of rats following acute dermal exposure to Novostron. **S9 File.** Plasma biochemical parameters of rats following acute dermal exposure to Novostron.
(ZIP)

S2 Text S5 File. Hematological parameters of rats following acute dermal exposure to Novostron. (ZIP)
